# Supramolecular Lipid Nanoparticles Based on Amine β-CD Host–Guest Lipids: Design, Mechanisms, and Biosafety

**DOI:** 10.3390/pharmaceutics17111410

**Published:** 2025-10-30

**Authors:** Pin Lv, Yamin Li, Gang Du, Jiawei Ding, Jiawei Zhou, Yuan Zhang, Huang Lin, Ming Yang, Chao Zhou, Bo Yang

**Affiliations:** 1 Faculty of Life Science and Technology, Kunming University of Science and Technology, Kunming 650500, China; lvpin@yaas.org.cn (P.L.); liyamin@kmust.edu.cn (Y.L.); dugang@kmust.edu.cn (G.D.); dingjiawei@kmust.edu.cn (J.D.); jiaweizhou@kmust.edu.cn (J.Z.); zhouchao@kmust.edu.cn (C.Z.); 2 Industrial Crop Research Institute, Yunnan Academy of Agricultural Sciences, Kunming 650225, China; zhangyuan@yaas.org.cn (Y.Z.); haungl@yssa.org.cn (H.L.); ymhemp@yaas.org.cn (M.Y.); 3 Yunnan Key Laboratory of Genetic Improvement of Herbal Oil Crops, Kunming 650225, China

**Keywords:** drug delivery, formulation studies, multi-stage assembly, kidney-targeting, acidic pH-responsive

## Abstract

**Background/Objectives:** Lipid nanoparticles (LNPs) have demonstrated notable clinical success as advanced drug delivery systems. However, the development of novel covalently bonded ionizable lipids faces substantial technical challenges, as their modification is difficult and they have a high molecular weight. To address this issue, we report the use of host–guest complexes in supramolecular chemistry as functional lipid motifs for constructing LNPs. **Methods:** Ionizable amine β-cyclodextrin (amine β-CD)-derived host–guest amphiphilic lipid molecules (HGLs) were designed for the construction of multi-stage assembly supramolecular LNPs (MSLNPs). The structure–function relationships and stability of MSLNPs were explored by screening eight types of amine β-CDs and varying the ratio of HGL to yolk phosphatidylcholine. Stability screening and molecular dynamics simulations were performed to clarify the self-assembly mechanisms and optimal formulations, followed by a systematic evaluation of delivery performance. **Results:** MSLNPs showed a high drug-loading efficiency (> 30%), a rapid-response release in acidic environments, and multi-pathway cellular uptake. In vivo delivery experiments using ethylenediamine β-CD-based MSLNPs in mice revealed no significant immunogenicity, no significant abnormalities in organs/tissues or their functions, a unique biodistribution pattern, and pronounced renal targeting. The successful development of MSLNPs with acidic pH-responsive control, a high delivery efficiency, and renal-targeting properties simplifies LNP preparation. **Conclusions:** This study offers novel insights into the design of simplified LNPs and the optimization of targeted delivery, with potential applications in renal disease therapy.

## 1. Introduction

Lipid nanoparticles (LNPs) have revolutionized drug delivery research in recent years, with their translational success exemplified by the COVID-19 mRNA vaccines and the landmark approval of Casgevy (exagamglogene autotemcel), which is the world’s first CRISPR/Cas9 gene-editing therapeutic, by late 2023 [1,2,3,4,5]. Although LNPs are broadly categorized as lipid-based nanocarriers, their architecture markedly differs from that of conventional vesicular liposomes. Modern LNPs designed for nucleic acid delivery typically adopt a non-vesicular, solid core structure, wherein nucleic acids are encapsulated and shielded within a hydrophobic lipid core, externally stabilized by a lipid monolayer and PEG–lipid conjugates [6,7,8]. The composition of LNPs has been refined through multiple developments, starting with the earliest liposomes composed of natural lipid molecules, polyethylene glycol lipids, and cholesterol. Examples include marketed formulations such as liposomal irinotecan and doxorubicin [9,10]. Current lipid delivery systems are predominantly constructed using chemically synthesized ionizable or cationic lipids. For instance, the first generation of mRNA vaccines for COVID-19 prophylaxis, Comirnaty (BNT162b), and Spikevax (mRNA-1273), leveraged the ionizable lipids ALC-0315 and SM-102, respectively. Both vaccines demonstrated high efficacy in preventing viral prevention [11,12]. Moreover, numerous researchers have discussed the use of modified cationic lipids, such as Alnylam’s Patisiran, which is used for treating hereditary transthyretin amyloidosis [13]. The research group led by Ming Wang developed LNPs with BAMEA-O16B, which can efficiently deliver Cas9 mRNA/sgRNA into liver cells, resulting in significant gene-editing effects [14]. Despite these advances, the development of covalently bonded ionizable lipids remains challenging, owing to complex synthesis, limited biodegradability, poor targetability, and suboptimal stability [15,16]. These limitations hinder the clinical translation of LNPs, particularly for renal-targeted drug delivery, where conventional LNPs struggle with rapid hepatic clearance, insufficient kidney accumulation, and lack of microenvironment-responsive release [17,18].

To overcome these challenges, we turned to supramolecular chemistry, proposing that dynamic, non-covalent host–guest complexes could serve as functional lipid motifs. This approach circumvents the synthetic complexity of covalent lipids by using well-defined, biocompatible building blocks like cyclodextrins (CDs) [19]. Dynamic reversible assemblies constructed through non-covalent interactions typically exhibit improved stability and drug-loading efficiency. Compared with covalent lipid molecules, the introduction of host–guest complexes from supramolecular chemistry as amphiphilic lipids in LNPs provides a feasible solution to existing problems [20]. The advantages of such frameworks stem from the independent functionalization of host and guest molecules, their strong interactions, and the stimulus-responsive nature of host–guest recognition. These characteristics make host–guest complexes highly suitable for the construction of LNP structures [21,22].

**Scheme 1:**
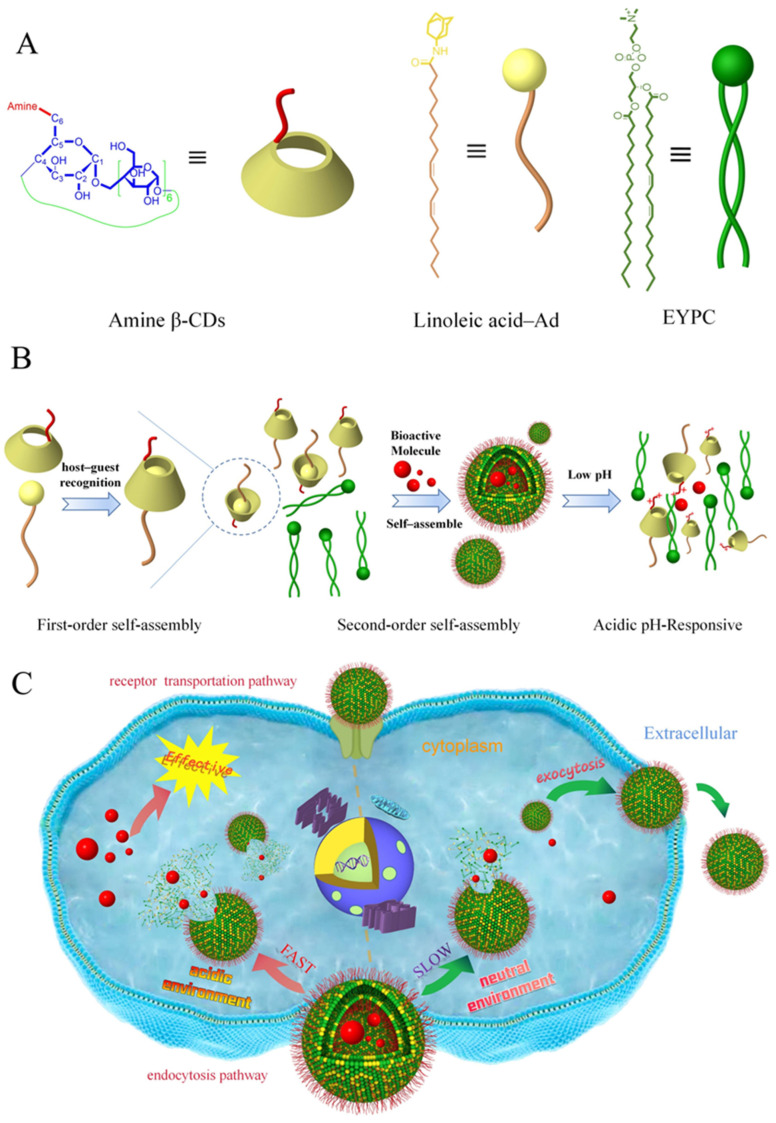
Schematic diagram of the MSLNP assembly and biological delivery process. (**A**) Chemical structures and representations of amine β-CDs, linoleic acid–Ad, and EYPC. (**B**) Schematic of multi-stage self-assembled amine-based β-CDs and pH-responsive drug-release mechanism. (**C**) Schematic of the MSLNP delivery pathway and controlled-release mechanism under different pH environments. EYPC, egg yolk phosphatidylcholine; β-CD, β-cyclodextrin.

Considering these aspects, we hypothesized that incorporating acidic pH-responsive amine β-CDs into LNP formulations could simplify their composition while achieving high delivery performance and enhanced controllability. We synthesized low-pH-responsive amine-modified β-CDs, which became protonated under acidic conditions to enhance their positive charge, and self-assembled with single-chain linoleic-acid-modified diamantane to form host–guest amphiphilic lipid molecules (HGLs). Using HGL as the main lipid molecule, we constructed multi-stage assembled supramolecular lipid nanoparticles (MSLNPs) and studied their composition, proportions, size, shape, and stability. Molecular dynamic (MD) simulations were performed to investigate the interaction mechanisms between the components of MSLNPs. We also investigated the optimal MSLNP formulation in terms of the drug encapsulation efficiency, in vitro drug-release performance, and delivery pathways. Cellular uptake experiments, transfection assays, and in vivo studies in mice were performed to systematically investigate the delivery performance of drug-loaded MSLNP systems, including cellular internalization pathways, delivery efficiency, in vivo expression profiles, and biosafety. These analyses further elucidated the mechanisms of action and dynamic processes of MSLNPs in vivo. The successful development of MSLNPs offers a valuable pathway toward a versatile delivery platform with kidney-targeting capabilities and a high clinical value (Scheme 1).

## 2. Materials and Methods

### 2.1. Materials and Instruments

Linoleic acid (CAS No. 60-33-3, MW = 280.45, 98% purity), β-CD (CAS No. 7585-39-9, MW = 1134.98, 95%+ purity), ethylenediamine (CAS No. 107-15-3, MW = 60.1, ≥99% purity), diethylenetriamine (CAS No. 111-40-0, MW = 103.17, 99% purity), triethylenetetramine (CAS No. 112-24-3, MW = 146.23, ≥97% purity), dimethylaminoethylamine (CAS No. 108-00-9, MW = 88.15, 99% purity), diethylethylenediamine (CAS No. 100-36-7, MW = 116.2, ≥99% purity), 1-(2-aminoethyl) pyrrolidine (CAS No. 7154-73-6, MW = 114.19, 98%+ purity), 4-(2-aminoethyl) morpholine (CAS No. 2038-03-1, MW = 130.19, 99%(GC-MS) purity), amantadine (Ad, CAS No. 768-94-5, MW = 151.25, 98%(HPLC) purity), Triton X-100 (CAS No. 9002-93-1, MW = 324.41, Biochemical), amikacin (CAS No. 37517-28-5, MW = 585.6, 95% purity), chlorpromazine (CAS No. 50-53-3, MW = 318.86, 95%+ purity), cyclosporine A (CAS No. 59865-13-3, MW = 1202.61, 99% purity), ethyl-3-(3-dimethylaminopropyl) carbodiimide (EDCI, CAS No. 7084-11-9, MW = 191.7, 97% purity), and 1-hydroxybenzotriazole (HOBT, CAS No. 2592-95-29, MW = 135.12, 99% purity) were purchased from the Tansoole platform (https://www.tansoole.com/). Egg yolk phosphatidylcholine (EYPC, CAS No. 8002-43-5, MW = 758.06, ≥99% (TLC) purity), 1,2-Dioctadecanoyl-sn-glycero-3-phophocholine (DSPC, CAS No. 816-94-4, MW = 790.15, 99% purity), and SM102 (CAS No. 2089251-47-6, MW = 710.17, 98% purity)) were purchased from AVT (Shanghai, China) Pharmaceutical Tech Co., Ltd., and pcDNA3.1-EGFP (pDNA_(EGFP)_) was purchased from Hunan Fenghui Biotechnology Co., Ltd. (Wuhan, China). Specific-pathogen-free-grade male BALB/c mice were purchased from Yunnan Besitel Biotechnology LLC (Kunming, China). Nuclear magnetic resonance (NMR) experiments were conducted using a Bruker Avance DRX spectrometer operating at 600 MHz, with tetramethylsilane (TMS) serving as the internal standard. The solvents included deuterium oxide (D_2_O, CAS No. 7789-20-0, MW = 20.03, (D 99.8%) purity), dimethyl sulfoxide-d6 (DMSO-d_6_, CAS No. 2206-27-1, MW = 84.17, (D 99.8%) purity), and deuterated chloroform (CDCl_3_, CAS No. 865-49-6, MW = 120.38, (D 99.8%) purity) were purchased from Aladdin Industrial Corporation (Shanghai, China). Mass spectrometric analyses were performed using an Agilent 6530 Accurate-Mass QTOF LC/MS system, equipped with a heated electrospray ionization source and operated in positive ionization mode.

All animal experiments were conducted with ethical approval. (Ethics Committee Name: Institutional Animal Care and Use Committee (IACUC) of Yunnan Besitel Biotechnology LLC (Kunming, China); approval code: BST-PZ-MICE-20240826-01; approval date: 26 August 2024).

### 2.2. Methods

#### 2.2.1. Synthesis of Amine β-CDs and Linoleic Acid–Ad

β-CDs were reacted with tosyl chloride (TsCl) in an alkaline aqueous medium, and subsequent purification yielded a white powder corresponding to 6-OTs-βCDs. These intermediates, as the substrate, were subsequently reacted with eight distinct amino compounds. Following purification, a light-yellow powder was obtained, representing amine β-CDs. Linoleic acid–Ad was prepared by condensation of linoleic acid and adamantylamine. Detailed experimental procedures, molar ratios, and characterization data are provided in the Supporting Information.

#### 2.2.2. Preparation of HGL as Linoleic Acid–Ad/Amine β-CD Inclusion Complex

HGL was synthesized using the suspension method. Initially, linoleic acid–Ad (0.03 mM) and amine β-CDs (0.01 mM) were dissolved in distilled water. The suspension was stirred at 20–25 °C for 48 h in the dark. Subsequently, any undissolved linoleic acid–Ad was filtered out using a 0.45 μm microporous membrane. The filtrate was vacuum dried at 50 °C for 24 h to yield the linoleic acid–Ad/amine β-CD inclusion complex (HGL).

#### 2.2.3. Preparation of Linoleic Acid–Ad/Amine β-CD Physical Mixture

Amine β-CDs and linoleic acid–Ad (molar ratio = 1:1) were thoroughly mixed by grinding in an agate mortar for 3 min. (During the grinding process, particular attention was paid to periodically scrape material from both the pestle and mortar walls to ensure comprehensive integration of the solid and liquid phases.) Subsequently, the mixture was dried in a vacuum oven at 50 °C for 24 h, resulting in the linoleic acid–Ad/amine β-CD physical mixture.

#### 2.2.4. General Methods

General characterization methods included determination of acid dissociation constant (pKa) values, construction of phase–solubility diagrams, scanning electron microscopy (SEM, SEM analysis was performed using a JEOL JSM 840 scanning electron microscope with samples sputter-coated with a gold layer for 60 s to ensure conductivity, and imaged at an accelerating voltage of 15 kV), transmission electron microscope (TEM, TEM analysis was conducted using a Tecnai G2 F30 S-TWIN microscope at an accelerating voltage of 200 kV, with samples prepared by depositing the solution onto copper grids and air-drying prior to imaging), particle size and zeta potential measurements (the particle size, size distribution (PDI), and zeta potential of the nanoparticles were characterized using a Zetasizer-Nano ZS90 (Malvern Panalytical, Malvern, UK). DLS measurements were performed at a set temperature with a 90° scattering angle in quartz cuvettes, while zeta potential was determined in quartz cuvettes at the same set temperature with automatic voltage selection and the Smoluchowski model. All samples were analyzed in triplicate using water’s optical constants for calculation), and NMR analysis (^1^H NMR and 2D ROESY NMR spectra were acquired on a Bruker Avance DRX spectrometer operated at 600 MHz and 298 K). Detailed procedures and parameters are provided in the Supporting Information.

#### 2.2.5. MD Simulations

MD simulations were performed using GROMACS 2022 software, applying the CHARMM General Force Field for small molecules and the TIP3P water model for solvation. A cubic water box sized 10 × 10 × 10 nm^3^ was constructed, ensuring a minimum buffer distance of 1.2 nm between the solute and box edges. The system was neutralized by adding counterions and randomly filled with 50 molecules each of EN-βCDs, linoleic acid–Ad, and EYPC (or DETA-βCDs/linoleic acid–Ad/EYPC = 42/42/63) to enable spontaneous self-assembly into nanoclusters. The simulation workflow included energy minimization using the steepest descent algorithm (50,000 steps) to eliminate steric clashes, followed by NVT equilibration with a Langevin thermostat at 300 K to stabilize temperature, and NPT equilibration using the Berendsen barostat at 1 bar to equilibrate pressure and density. A production run of 200 ns under the NPT ensemble was then performed for trajectory sampling. Electrostatic interactions were calculated using the particle mesh Ewald method with Coulombic and van der Waals cutoff distances set to 1.0 nm. Non-bonded interactions were truncated at 10 Å and updated every 10 steps. Trajectories were saved every 10 ps for analysis. Visualization and structural interpretation of the simulation results were performed using GROMACS built-in tools and Visual Molecular Dynamics (VMD, Version 1.9.4, University of Illinois at Urbana-Champaign, USA).

#### 2.2.6. Preparation of MSLNPs

Considering experimental requirements, a specific concentration of HGL was dissolved in 5 mL of water to serve as the aqueous phase, while a specific concentration of EYPC was dissolved in 1 mL of ethanol to serve as the organic phase. The total molar amount was maintained at 1.0 mmol (*n*_(HGL)_ + *n*_(EYPC)_ = 1.0 mmol). The two phases were passed through a microfluidic device equipped with a Y-shaped chip at a total flow rate of 12 mL/min, and the reaction solution was collected. The reaction solution was then dialyzed (MWCO = 5 KDa) to remove ethanol and other solvents, ultimately yielding MSLNPs.

#### 2.2.7. Preparation of Drug-Loaded MSLNP Systems

Considering experimental requirements, a specific concentration of HGL was dissolved in 5 mL of water to serve as the aqueous phase, while a specific concentration of EYPC was dissolved in 1 mL of ethanol to serve as the organic phase. The total molar amount was maintained at 1.0 mmol (*n*_(HGL)_ + *n*_(EYPC)_ = 1.0 mmol). A total of 5.0 mmol of drug molecules was weighed. Hydrophilic drug molecules were dissolved in the aqueous phase, while hydrophobic drug molecules were dissolved in the organic phase. The aqueous and organic phases were then passed through a microfluidic device equipped with a Y-shaped chip at a total flow rate of 12 mL/min, and the reaction solution was collected. Finally, the reaction solution was dialyzed (MWCO = 5 KDa) to remove ethanol, free drugs, and other components, ultimately yielding drug-loaded MSLNP systems. The preparation methods for all other types of LNPs also followed this procedure, with the total lipid amount fixed at 1 mmol.

#### 2.2.8. Characterization of MSLNPs

The characterization of MSLNPs, including encapsulation efficiency, drug-release, and demulsification profiles, along with detailed methodological descriptions, is provided in the Supporting Information.

#### 2.2.9. Evaluation of In Vitro and In Vivo Delivery Performance of MSLNPs

The in vitro and in vivo delivery performance of MSLNPs was analyzed through cellular uptake experiments under varying conditions and assessment of physiological/biochemical data for mice. Detailed experimental protocols and parameters are presented in the Supporting Information.

## 3. Results and Discussion

### 3.1. Characterization of Synthesized Amine β-CDs and Linoleic Acid–Ad

**Scheme 2:**
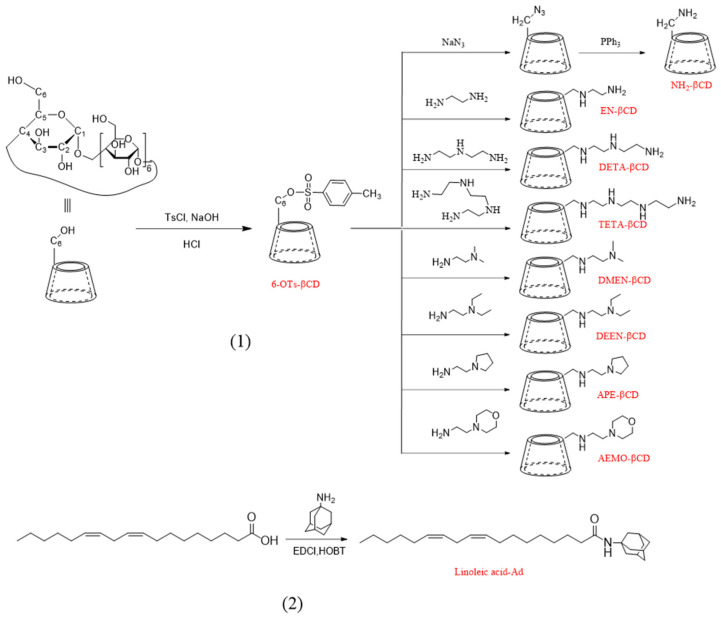
Synthesis routes of (1) amine β-CDs and (2) linoleic acid–Ad. Ad, amantadine; β–CD, β–cyclodextrin.

The lipid molecules for the LNPs were synthesized by employing β-CDs of varying nitrogen-branching degrees as the head groups and adamantane-grafted linoleic acid as the hydrophobic tails, which exploits the strong host–guest affinity between adamantane and β-CDs. Cholesterol was added to adjust the fluidity of the LNPs. The synthetic schemes for amine β-CDs and linoleic acid–Ad are shown in Scheme 2. Eight types of amino-functionalized cyclodextrins were synthesized: amino-βCDs (NH_2_-βCDs), ethylenediance-βCDs (EN-βCDs), diethylenetriamine-βCDs (DETA-βCDs), and triethylenetetramine-βCDs (TETA-βCDs). Additionally, we synthesized four new compounds including N,N-dimethylaminoethylamine-βCDs (DMEN-βCDs), N,N-diethylethylenediamine-βCDs (DEEN-βCDs), 1-(2-aminoethyl) pyrrolidine-βCDs (AEP-βCDs), and 4-(2-aminoethyl) morpholine-βCDs (AEMO-βCDs). Characterization data for these novel amino-functionalized cyclodextrins are presented in Figures S1–S4.

To select the hydrophobic tails for the HGL, we considered single-chain fatty acid molecules. Linoleic acid was identified as the most suitable candidate for several reasons: first, it is an essential fatty acid in humans, offering high safety; second, it is readily available and economically viable, with significant concentrations found in sources such as hemp seeds and flaxseeds; and third, it is a monounsaturated fatty acid with a single chain of 18 carbon atoms, providing an enhanced cone-shaped structure and increased cross-section in the tail region compared with saturated fatty acids. Research indicates that unsaturated tail chains like that of linoleic acid, facilitate intracellular degradation and drug release from LNPs [23]. In accordance with our experimental design, the guest molecules were synthesized from linoleic acid and adamantane (Figure S5).

### 3.2. Determination of pKa Values

pKa is a critical physicochemical property of the ionizable head group in LNPs, as it determines the ionization behavior and surface charge of LNPs, which greatly influence their stability, efficacy, toxicity, and controlled-release capabilities. Notably, environmental factors such as ionic strength, dielectric constant, and nanoparticle size/shape lower the observed pKa compared with the monomeric form.

**Figure 1:**
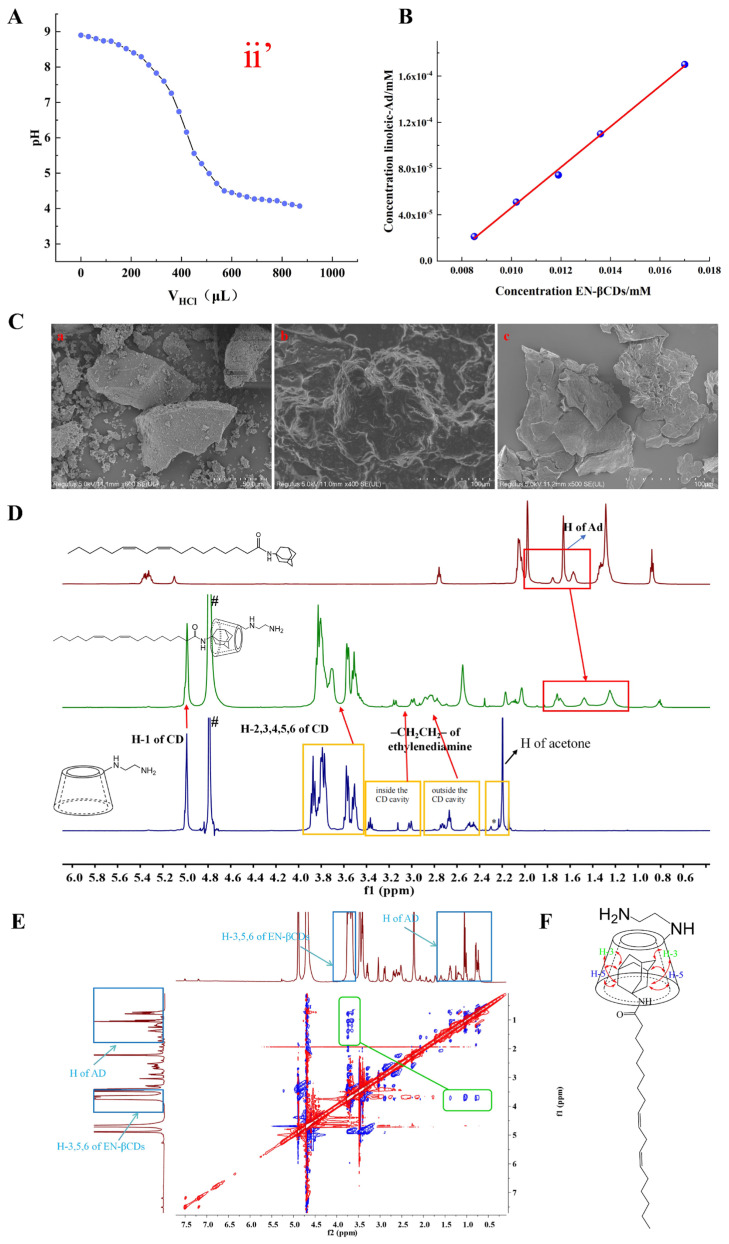
Characterization of physicochemical properties of HGL. (**A**) pKa data for (ii’) HGL_(EN-βCDs)_; (**B**) phase–solubility diagram: EN-βCD/linoleic acid–Ad interaction dynamics, Concentration_(linoleic-Ad)_ = (−1.29357 × 10^−4^ ± 6.18438 × 10^−6^) + (0.01754 ± 4.91425 × 10^−4^) × Concentration_(EN-βCDs)_, R^2^ = 0.99765; (**C**) SEM images of HGL and its constituent components (a) EN-βCDs, (b) physical mixture of EN-βCDs with linoleic acid–Ad, and (c) EN-βCDs/linoleic acid–Ad inclusion complex; (**D**) ^1^H NMR spectra (from top to bottom) are linoleic acid–Ad, EN-βCDs/linoleic acid–Ad inclusion complex in D_2_O, and EN-βCDs; “#” indicates solvent peaks and “*” denotes minor impurities from the substrate; (**E**) 2D ROESY NMR spectra, showing intermolecular interactions of the EN-βCDs/linoleic acid–Ad inclusion complex; (**F**) possible inclusion mode of linoleic acid–Ad/EN-βCD complex. Ad, amantadine.

The number and position of -NH- groups in the molecule influence their binding efficiency with hydrogen protons, thereby leading to differences in the molecular pKa values. To identify suitable molecules, we measured the pKa values of the amine β–CDs and corresponding HGLs, and the results are shown in Figure 1A and Figure S6. The pKa values of the eight amine β–CDs exhibited slight differences, with TETA–βCDs showing the highest pKa (7.699) and NH_2_–βCDs the lowest (6.549). Whereas the pKa values of all eight amine β–CDs were close to the target value, making them potential ionizable head groups, the values decreased in the HGL formed with linoleic acid–Ad. Only the HGL prepared from EN–βCDs exhibited a pKa within the ideal range of 6–7, while the pKa values of HGLs derived from six other β–CD derivatives—DETA–βCDs (7.213), TETA–βCDs (7.522), DMEN–βCDs (7.108), DEEN–βCDs (7.251), AEP–βCDs (7.166), and AEMO–βCDs (7.536)—remained close to the optimal range. Notably, pKa values further decreased upon assembly into MSLNPs, qualifying these compounds as viable candidates for constructing lipid molecules in MSLNP formulations.

### 3.3. Characterization of HGL Prepared from Amine β-CDs and Linoleic Acid–Ad

The structural characteristics of the seven HGLs were analyzed using multiple analytical methods, including phase–solubility analysis, SEM, ^1^H NMR, and two-dimensional rotating-frame Overhauser effect spectroscopy (2D ROESY NMR). All HGL compounds exhibited similar structural profiles. Thus, the HGL formed with EN–βCDs was selected as a representative case for detailed analysis, as shown in Figure 1B–E. The phase–solubility diagrams of EN–βCDs and linoleic acid–Ad are presented in Figure 1B and Figure S7 and Table S1.

The solubility of linoleic acid–Ad increased linearly with the addition of EN–βCDs, following the typical A_L_-type curve described by Higuchi and Connors’s theory. This further confirmed the formation of a 1:1 inclusion complex between the EN–βCDs and linoleic acid–Ad. The association constant (*Ks*) for the interaction between the two entities, determined using Equation (1), was 300 M^−1^, indicating substantial host–guest interaction.

(1) $$Ks=\frac{Slope}{S0(1-Slope)}$$

where *Slope* represents the slope of the phase-solubility diagram, and *S*_0_ is the solubility of free linoleic acid–Ad (5.95 × 10^−5^ mol/L at 25 °C).

SEM analysis (Figure 1C) was performed on EN-βCDs, linoleic acid–Ad, their inclusion complex, and their physical mixtures. Linoleic acid–Ad, being an oily substance, appeared completely dark under the scanning electron microscope. Therefore, its image is not displayed. Image (a) is EN-βCDs, showing an irregular three-dimensional block structure with a porous surface. Image (b) presents the physical mixture of EN-βCDs and linoleic acid–Ad. Due to the inability of linoleic acid–Ad, it could not be properly imaged, so the resulting micrograph shows indistinct boundaries. Image (c) corresponds to the EN-βCDs/linoleic acid–Ad inclusion complex, which exhibits a distinctly irregular block structure compared to EN-βCDs. These morphological inconsistencies provide supporting evidence for the formation of an inclusion complex rather than a simple physical mixture.

Following the stacking of the ^1^H NMR spectra for the guest, inclusion complex, and host, proton peaks corresponding to adamantane were observed in the spectrum of the inclusion complex, indicating its successful formation (Figure 1D). Furthermore, 2D NMR spectra displayed clear NOE cross-peaks between the adamantane portion of linoleic acid–Ad and the cavity of CDs (Figure 1E). The combination of phase–solubility analysis, SEM, and NMR data mutually verified the formation of the EN-βCDs with linoleic acid–Ad inclusion complex and suggested a possible inclusion mode (Figure 1F).

### 3.4. Formulation Study of MSLNPs

**Figure 2:**
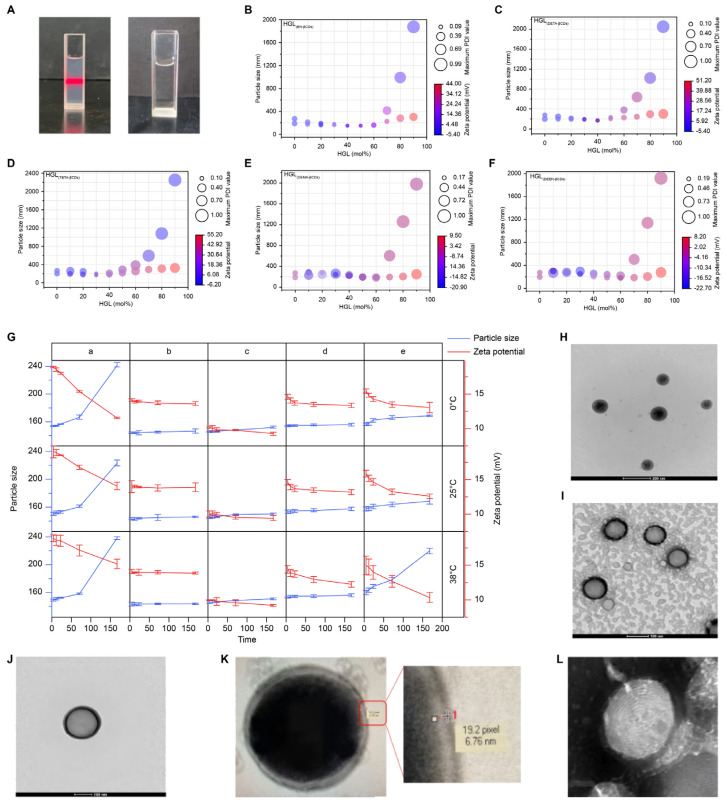
Characterization of physicochemical properties of MSLNPs. (**A**) Laser irradiation images of freshly prepared MSLNPs solution (left) and dialyzed MSLNP solution (right); bubble plot of MSLNP particle size, zeta potential, and DPI data pertaining to different HGL/EYPC ratios (bubble diameter represents the PDI, and color scale maps to the zeta potential); (**B**) HGL_(EN-βCDs)_; (**C**) HGL_(DETA-βCDs)_; (**D**) HGL_(TETA-βCDs)_; (**E**) HGL_(DMEN-βCDs)_; (**F**) HGL_(DEEN-βCDs)_. (**G**) MSLNP particle sizes (different HGL/EYPC ratios) and zeta potential at different temperatures and time. TEM images of (**H**) MSLNPs_(DETA-βCDs)_; (**I**–**L**) MSLNPs_(EN-βCDs)_. HGL, host–guest amphiphilic lipid molecules.

Following the successful synthesis of HGL prepared with EN-βCDs and linoleic acid–Ad (HGL_EN-βCDs_), microfluidic technology was employed to examine the formulation of MSLNPs. Initially, MSLNPs was prepared using 100% HGL. The collected reaction mixture exhibited the Tyndall effect upon laser irradiation, indicating the presence of colloidal structures rather than a true solution. Remarkably, upon post-dialysis purification, the reaction mixture no longer exhibited the Tyndall effect under laser illumination, as depicted in Figure 2A.

To elucidate the mechanism underlying the cessation of the Tyndall effect, the experiment was repeated under identical parameters. The reaction mixture was divided into three aliquots: (a) analyzed immediately by dynamic light scattering (DLS); (b) left undisturbed in a cuvette for 6 h prior to DLS analysis, and (c) subjected to dialysis for 6 h before DLS analysis (c). The results are shown in Figure S8. Sample (a) exhibited a particle diameter of 372 ± 25 nm and polydispersity index (PDI) of 0.4 to 0.5, indicating the presence of nanoparticle structures, albeit with potential aggregation, as indicated by sizes > 220 nm. Conversely, Samples (b) and (c) exhibited particle diameters exceeding 1000 nm, suggesting the dissolution of nanoscale particles within the reaction mixture.

Given these results, we hypothesize that the instability of the constructed MSLNPs may be attributable to size mismatch between the larger head group of EN-βCDs (size > 7 Å) and smaller hydrophobic tails (size < 1 Å) within HGLs [24]. Furthermore, electrostatic repulsion between EN-βCD molecules likely leads to insufficient intermolecular forces among HGL units, contributing to the instability of the formed MSLNPs. For this purpose, we introduced EYPC as an auxiliary, slightly negatively charged phospholipid to stabilize the MSLNP structure. This choice was based on its high safety profile and cost-effectiveness. To ascertain the optimal formulation of MSLNPs, LNPs were engineered using varying ratios of HGL to EYPC, with the EYPC content increasing from 10 mol% to 100 mol%. The particle diameter and zeta potential were evaluated under different conditions, and the findings are presented in Tables S2–S8 and Figure 2B–F.

Following rapid dialysis to remove ethanol, the reaction mixture was divided into two portions: one was immediately analyzed by DLS to determine the particle size and zeta potential, while the other was diluted tenfold prior to measurement. As indicated in Tables S3–S8, HGL_(AEP-βCDs)_ and HGL_(AEMO-βCDs)_ formed nanoparticle structures only at higher EYPC contents. However, these formulations failed to maintain their nanoarchitectures after tenfold dilution (particle size > 200 nm, PDI > 0.3, or undetectable), rendering them unsuitable for MSLNP fabrication. In contrast, for the other five experimental groups, the particle size initially decreased with increasing EYPC content in the undiluted series. However, beyond a certain ratio, further EYPC addition did not reduce particle size; in fact, slight increase was observed. To intuitively screen MSLNP formulations with optimal particle size, zeta potential, and dilution resistance, bubble plots were generated to analyze data from HGL_(EN-βCDs)_, HGL_(DETA-βCDs)_, HGL_(TETA-βCDs)_, HGL_(DMEN-βCDs)_, and HGL_(DEEN-βCDs)_. The *x*-axis was the molar ratio of HGL, and *y*-axis was the particle size; bubble diameter represented the PDI, and color gradient denoted the zeta potential. Data from both undiluted and tenfold diluted groups were overlaid for comparative analysis (Figure 2B–F).

Stability was assessed by comparing the bubble characteristics (color, size, and centroid position) of diluted and undiluted groups, with closer alignment indicating higher structural integrity. This approach identified robust formulations: HGL_(EN-βCDs)_ (40%, 50%, 60%), HGL_(DETA-βCDs)_ (30%, 40%, 50%), HGL_(TETA-βCDs)_ (30%, 40%), HGL_(DMEN-βCDs)_ (20%, 30%, 40%), and HGL_(DEEN-βCDs)_ (10%, 20%). Further refinement based on in vivo delivery criteria (size < 200 nm, zeta potential +10 to +20 mV) narrowed the selection to the five top candidates: HGL_(EN-βCDs)_ (40%, 50%, 60%), HGL_(DETA-βCDs)_ (40%), and HGL_(TETA-βCDs)_ (30%). These formulations maintained nanoscale stability under physiological dilution, demonstrating strong potential for advancing lipid-based nanocarrier design.

The stability of various MSLNP formulations was assessed under different temperatures and storage times to simulate their transportation and storage conditions. Based on preliminary screening results, five formulations were selected for evaluation: (a) 60 mol% HGL_(EN-βCDs)_ + 40 mol% EYPC; (b) 50 mol% HGL_(EN-βCDs)_ + 50 mol% EYPC; (c) 40 mol% HGL_(EN-βCDs)_ + 60 mol% EYPC; (d) 40 mol% HGL_(DETA-βCDs)_ + 60 mol% EYPC; (e) 30 mol% HGL_(TETA-βCDs)_ + 70 mol% EYPC. The prepared MSLNPs were stored at 0 °C, 25 °C, and 38 °C for 6, 12, 24, and 72 h, as well as 7 d. Stability was assessed by measuring the particle size, zeta potential, and PDI at each time point. The results are presented in Figure 2G and Table S9.

Across all tested groups, PDI values remained below 0.20, demonstrating that MSLNPs with these three compositional ratios could retain stable nanoparticle states under varying environmental conditions. In Group (a), the MSLNPs exhibited minimal increase in particle size within the first 72 h but significant enlargement thereafter (≈1.5 times the initial size), accompanied by a gradual decline in zeta potential. This suggests partial fusion and aggregation of nanoparticles over time under all tested conditions for this formulation.

In contrast, Groups (b), (c), and (d) displayed negligible changes in particle size and zeta potential across storage durations and temperatures, with no statistically significant differences observed, indicating high stability. Notably, Group (c) exhibited a weakly positive zeta potential (<10 mV), which is generally considered insufficient for efficient cellular uptake. In contrast, Groups (b) and (d) maintained zeta potentials between 13 mV and 15 mV under all conditions, which is a range favorable for enhanced cellular internalization. For Group (e), particle size and zeta potential remained stable at 0 °C and 25 °C. However, at 38 °C, particle size increased rapidly after 24 h, exceeding 200 nm by 72 h, rendering this formulation unsuitable for cellular delivery. Overall, in this systematic screening, MSLNPs formulated with 50 mol% HGL_(EN-βCDs)_ and 40 mol% HGL_(DETA-βCDs)_, combined with their corresponding EYPC ratios, demonstrated superior physicochemical properties (optimal size and zeta potential) for drug delivery and were thus selected as candidates for further investigation into advanced lipid-based nanocarrier design.

### 3.5. Morphological Study of MSLNPs

TEM was employed to examine the morphological characteristics of the two MSLNP formulations, specifically 50 mol% HGL_(EN-βCDs)_ and 40 mol% HGL_(DETA-βCDs)_ (Figure 2H–J). Figure 2H,I display the TEM images of MSLNPs_(DETA-βCDs)_ and MSLNPs_(EN-βCDs)_, respectively. Both formulations exhibited regular circular morphologies with diameters of 140–160 nm, consistent with the DLS observations. Additionally, an isolated nanoparticle field of view was identified in MSLNPs_(EN-βCDs)_ during TEM imaging, as shown in Figure 2J. Upon magnification, a transparent band approximately 6.76 nm thick was observed within the shell of the MSLNPs, appearing as two concentric circles (Figure 2K). This feature likely reflected the tight binding between the head group portions of EN-βCDs and EYPC, which appeared denser compared with their tail counterparts. Further magnification revealed distinct fingerprint patterns (Figure 2L), which is characteristic of LNP morphology. In summary, TEM results confirmed the successful preparation of MSLNPs.

### 3.6. MD Simulations of MSLNPs

**Figure 3:**
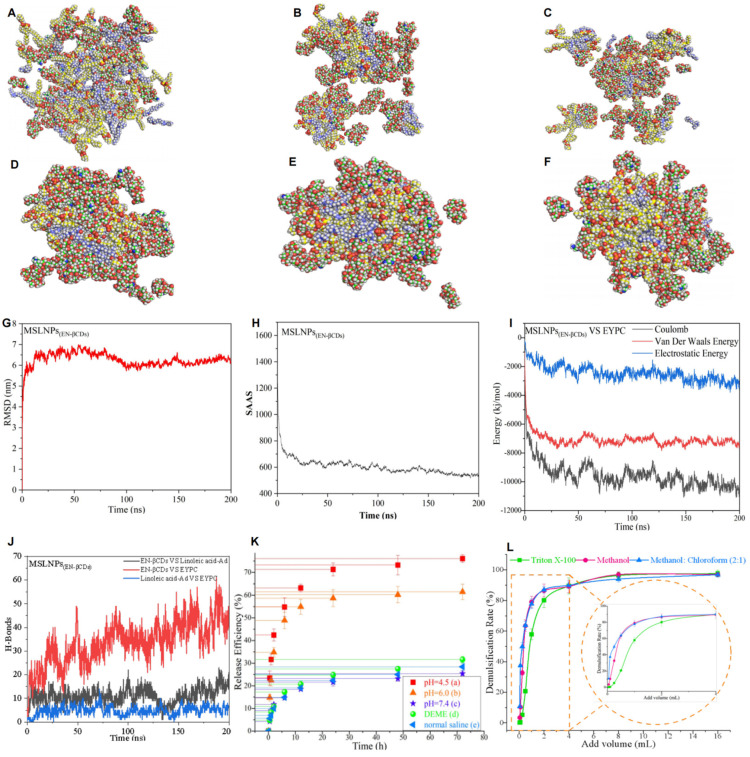
Assembly mechanism of MSLNPs and drug-release experiments under different environments. (**A**–**J**) Self-assembly molecular dynamics simulations of MSLNPs_(EN-βCDs)_. (**A**–**F**) Nanocluster formation process of EN-βCDs, linoleic acid–Ad, and EYPC, with image frames captured at intervals of 40 ns (C of βCDs, C of linoleic acid–Ad, and C of EYPC atoms are marked by green, blue, and yellow spheres, respectively); (**G**) RMSD of all atoms in MSLNPs_(EN-βCDs)_ system as a function of simulation time; (**H**) time-dependent changes in solvent-accessible surface area (SASA) in the MSLNPs_(EN-βCDs)_ systems during simulation; (**I**) time-dependent changes in electrostatic energy, van der Waals energy, and interaction energy among components of MSLNPs_(EN-βCDs)_ (HGL ↔ EYPC); (**J**) time-dependent changes in the number of intermolecular hydrogen bonds in the MSLNPs_(EN-βCDs)_. (**K**,**L**) Drug-release profiles of MSLNPs_(EN-βCDs)_: (**K**) under different environmental conditions; (**L**) with different demulsifiers. MSLNPs, multi-stage assembly supramolecular lipid nanoparticle.

MD simulations were performed to analyze the self-assembly processes of amine β-CDs, linoleic acid–Ad, and EYPC. Results for the EN-βCDs and DETA-βCDs groups are presented in Figure 3A–J and Figure S9, respectively. Given the similar results across groups, the EN-βCDs group was selected as a representative case for detailed analysis. To visualize the dynamic progression of continuous molecular self-assembly in the MSLNP_(EN-βCDs)_ system, one molecular conformation frame was extracted every 40 ns as a representative structure (Figure 3A–F). The results showed that, driven by intermolecular interactions, molecules in the MSLNP system gradually accumulated over simulation time, ultimately forming a stable nanocluster structure. The root mean square deviation (RMSD) of the system exhibited minor fluctuations during the initial 30 ns of the simulation (Figure 3G). After 100 ns, the amplitude of RMSD variations decreased, indicating that the system had reached equilibrium and was stabilized. As molecular self-assembly proceeded, solvent-exposed regions gradually decreased. Therefore, the solvent-accessible surface area (SASA) was used to evaluate the compactness of the supramolecular LNPs (Figure 3H). The SASA significantly decreased during the initial simulation stage (0–25 ns) and stabilized thereafter, demonstrating that EN-βCDs/linoleic acid–Ad and EYPC components formed tight nanoclusters through self-assembly, reducing the number of atoms exposed to the solvent. To investigate the forces driving self-assembly, the intermolecular electrostatic energy, van der Waals energy, and interaction energy between HGL and EYPC during MD simulations were statistically analyzed (Figure 3I). Over 200 ns, the average electrostatic energy was –2466.34 ± 523.18 kJ/mol, average van der Waals energy was –7080.32 ± 475.52 kJ/mol, and average interaction energy was –9546.67 ± 895.09 kJ/mol. Thus, van der Waals interactions dominated the self-assembly process, while electrostatic interactions played a secondary role.

As hydrogen bond counts can reflect the overall binding strength and resilience of MSLNPs_(EN-βCDs)_, the number of intermolecular hydrogen bonds formed during MD simulations was quantified (Figure 3J): EN-βCDs ↔ Linoleic acid–Ad (0–23, average of 10.98 ± 3.35); EN-βCDs ↔ EYPC (0–61, average of 33.77 ± 9.17); linoleic acid–Ad ↔ EYPC (0–14, average of 4.86 ± 1.90). The hydrogen bond counts between EN-βCDs and linoleic acid–Ad and those between linoleic acid–Ad and EYPC remained relatively stable throughout the 200 ns simulation. In contrast, hydrogen bonds between EN-βCDs and EYPC increased significantly over time, with their average count exceeding the sum of hydrogen bonds between other component pairs. This indicates that within the hydrogen bond network, interactions between the CD head groups of HGL and EYPC played a dominant role. The increasing number of hydrogen bonds over time resulted in a more tightly packed lipid layer structure in MSLNPs_(EN-βCDs)_. Simultaneously, these findings demonstrate the importance of introducing EYPC into the MSLNPs_(EN-βCDs)_ formulation for achieving optimal nanostructure and physicochemical properties.

A comparison of the average hydrogen bond counts between the two formulations revealed that the EN-βCDs group, with a higher molar ratio, exhibited a greater number of hydrogen bonds than the DETA-βCDs group. This indicates that MSLNPs_(EN-βCDs)_ possess enhanced stress resistance compared with MSLNPs_(DETA-βCDs)_. Consequently, the MSLNP formulation with 50 mol% HGL_(EN-βCDs)_ was selected for delivery studies.

### 3.7. Encapsulation Efficiency and Drug-Loading Capacity of MSLNPs_(EN-βCDs)_

The ultimate objective of developing novel delivery systems is to achieve efficient drug delivery within biological systems, with the delivery efficiency significantly affected by the drug-loading capacity of the carrier. LNPs can effectively encapsulate both hydrophilic molecules (such as mRNA, pDNA, and Rhodamine B) and hydrophobic molecules (such as paclitaxel and cisplatin). In this study, Rh B (hydrophilic), pDNA_(EGFP)_ (hydrophilic, negative charge), and cannabidiol (CBD, hydrophobic) were selected as model drugs. As indicated in Table 1 and Table S10, Figures S10 and S11, the encapsulation efficiency of MSLNPs_(EN-βCDs)_ was 33.99% for Rh B and 53.04% for CBD, and the drug-loading capacity was 21.87% for Rh B and 18.50% for CBD. Thus, MSLNPs_(EN-βCDs)_ exhibited a higher drug-loading capacity compared with conventional liposomes and LNPs (the LNPs_(SM102)_ were composed of DSPC/SM102/Chol/PEG at a molar ratio of 10/50/38.5/1.5 mol%, which was modeled after a classic LNP formulation). A comparison was conducted between LNPs_(SM102)_ and MSLNPs_(EN-βCDs)_ at an N/P ratio of 5 (with the same molar amount of lipid molecules in MSLNPs_(EN-βCDs)_ as in LNPs), using negatively charged hydrophilic pDNA_(EGFP)_ (Table 1). The results indicate that due to their positive surface charges, both LNPs_(SM102)_ and MSLNPs_(EN-βCDs)_ effectively encapsulate pDNA_(EGFP)_, resulting in high encapsulation efficiency and drug-loading capacity. In contrast, the unmodified LNP group showed no significant improvement. Moreover, owing to its stronger positive charge, the MSLNPs_(EN-βCDs)_ group demonstrated higher encapsulation efficiency and drug-loading capacity than the LNPs_(SM102)_ group.

Moreover, compared to conventional LNPs and LNPs_(SM102)_, MSLNPs_(EN-βCDs)_ contain a large number of hydrophilic EN-βCDs (derived from HGLs), which form a hydrated layer that incorporates water molecules, enabling excellent encapsulation of water-soluble cargo. Similar performance was observed for LNPs, LNPs_(SM102)_, and MSLNPs_(EN-βCDs)_ with hydrophobic drugs.

Moreover, MSLNPs_(EN-βCDs)_ contain a large number of hydrophilic EN-βCDs (from HGLs), which form a hydrated layer containing water molecules, enabling excellent encapsulation of water-soluble cargo. Consequently, MSLNPs_(EN-βCDs)_ demonstrated a higher loading capacity for water-soluble molecules compared with hydrophobic molecules.

### 3.8. Drug-Controlled-Release Capability and Demulsification Effect of MSLNPs_(EN-βCDs)_

In vitro drug-controlled-release experiments provide an in-depth understanding of drug-release behavior, help optimize formulation design, and are significant for establishing quality standards, predicting clinical outcomes, and evaluating stability. Rh B was used as a model molecule to evaluate the drug-release profiles from MSLNPs_(EN-βCDs)_ under different media and pH conditions, as shown in Figure 3K,L. Figure 3K displays the release profiles of Rh B from MSLNPs_(EN-βCDs)_ under different pH conditions (graphs (a)–(c)). Drug release was accelerated at pH 4.5, mimicking the acidic environment of organelles such as lysosomes and the endoplasmic reticulum, and at pH 6.0, which is representative of the acidic conditions typical around tumor tissues. During the initial 6 h interval, approximately 50% of the drug was released (54.8% at pH 4.5 and 48.9% at pH 6.0). Following this period, the release rate decreased, stabilizing at 76.1% and 61.5% for pH 4.5 and 6.0. This behavior can be attributed to the increased protonation of the -NH- groups in the amine β-CDs within the HGLs under acidic conditions, which disrupts the balance of intermolecular forces between HGL and EYPC. Consequently, this leads to the disintegration of MSLNPs_(EN-βCDs)_ and synchronous release of the model drug. As the pH decreases, both the release rate and cumulative release amount increase significantly. In contrast, under neutral conditions, MSLNPs_(EN-βCDs)_ maintain a stable nanostructure, enabling the model drug to remain securely encapsulated without leakage. These findings demonstrate the low-pH-responsive properties of MSLNPs_(EN-βCDs)_.

Conversely, at a neutral pH of 7.4, representing the blood environment, a pronounced decline was observed in both the release rate and total drug release, quantified at 25.4%. This attenuated release pattern aligned with stability evaluations of the MSLNP_(EN-βCDs)_ particles. Furthermore, as demonstrated in Figure 3K (graphs (d) and (e)), the drug-release quantities in Dulbecco’s Modified Eagle Medium and normal saline solutions were notably low, consistent with the results at pH 7.4. These findings underscore the stability of MSLNPs_(EN-βCDs)_ throughout cellular experiments and the formulation of MSLNPs_(EN-βCDs)_ injectable solutions, ensuring minimal drug leakage and thereby enhancing the accuracy of the experimental data obtained.

Various demulsifiers, each with distinct demulsification mechanisms, were added to the MSLNPs_(EN-βCDs)_ solution. The susceptibility and disintegration characteristics of MSLNPs_(EN-βCDs)_ in response to these demulsifiers were investigated by quantifying the resultant drug-release profiles, as illustrated in Figure 3L. The destabilizing agents operate via distinct mechanisms. Specifically, Triton X-100 intercalates into the lipid bilayer, disrupting the hydrophobic and van der Waals interactions between lipid molecules to destabilize the LNPs and release the cargo. Methanol, in contrast, acts as a polar protic solvent that disrupts the ordered lipid packing, thereby achieving the same goal. In contrast, chloroform, a nonpolar solvent, when used in conjunction with methanol, can alter the solubility parameters of the liposomal membrane, promoting the disintegration of the liposomal structure and enhancing the release of encapsulated drugs or other substances [25,26]. The results of our analysis highlighted the critical influence of demulsifiers on the structural stability of MSLNPs_(EN-βCDs)_. The demulsification rate of the methanol/chloroform mixture was higher than that of methanol alone, likely because HGL and EYPC are amphiphilic and exhibit greater solubility in a polar–nonpolar solvent mixture. Reduced solubility led to decreased interactive forces between HGL and EYPC, resulting in the disruption of the nanostructure. Additionally, at low dosages (less than 0.5 mL), Triton X-100 did not demonstrate effective demulsification. This may be ascribed to the abundance of hydroxyl groups on the outer surface of the cyclodextrin molecules in HGL, which formed hydrogen bonds with both EYPC and Triton X-100, establishing a temporary equilibrium. As the concentration of Triton X-100 increased, its demulsification efficacy approached that of the methanol/chloroform combination. In conclusion, contact with demulsifiers must be rigorously prevented during the preparation, storage, and application of MSLNPs_(EN-βCDs)_ to ensure quality and safety.

### 3.9. Delivery Performance of MSLNPs_(EN-βCDs)_ In Vitro

**Figure 4:**
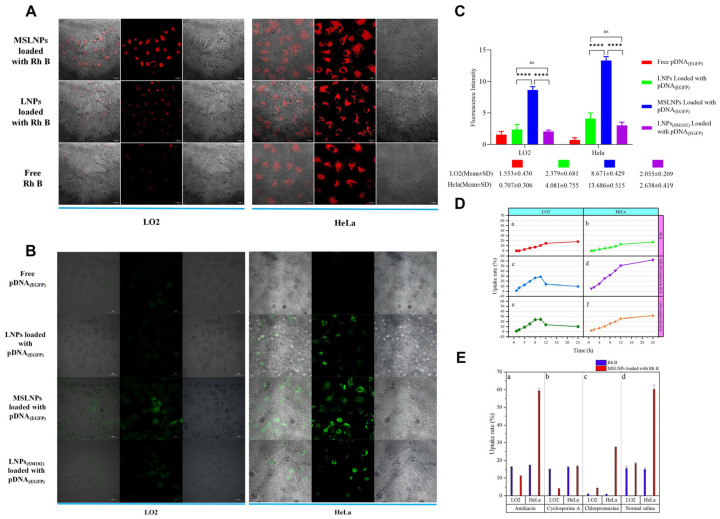
Uptake results of MSLNPs under different in vitro conditions. (**A**) Cellular uptake of free Rh B, LNPs loaded with Rh B, and MSLNPs loaded with Rh B in LO2 and HeLa cells (Rh B: 3.333 × 10^−4^ mol/L, *λ*ex = 514 nm, *λ*em = 585 ± 20 nm; scale bars are 20 μm); (**B**) cellular uptake of free pDNA_(EGFP)_, LNPs loaded with pDNA_(EGFP)_, LNPs_(SM102)_ loaded with pDNA_(EGFP)_, and MSLNPs loaded with pDNA_(EGFP)_ in LO2 and HeLa cells (pDNA_(EGFP)_: 2.5 ng, *λ*ex = 395 nm, *λ*em = 509 nm; scale bars are 20 μm). (**C**) Fluorescence intensity analysis of free pDNA_(EGFP)_, LNPs loaded with pDNA_(EGFP)_, LNPs_(SM102)_ loaded with pDNA_(EGFP)_, and MSLNPs loaded with pDNA_(EGFP)_ in LO2 and HeLa cells (**** *p* < 0.0001); (**D**) comparison of uptake rates of MSLNP system, LNP system, and free Rh B in different cell lines; (**E**) comparison of uptake efficiency of MSLNP system and free Rh B in different cell lines under various endocytosis inhibitor conditions. LNP, lipid nanoparticle.

Cellular uptake experiments were conducted to explore the interaction between cells and MSLNPs_(EN-βCDs)_. Rh B (or pDNA_(EGFP)_)- (the sequence structure is shown in Figure S12) loaded MSLNPs_(EN-βCDs)_ were added to LO2 and HeLa cells, respectively, and cell uptake was observed via confocal laser scanning microscopy (CLSM), as shown in Figure 4A,B. It is widely recognized that LO2 and HeLa cell lines do not inherently produce strong autofluorescence. However, when treated with Rh B, both cell groups exhibited faint red fluorescence under CLSM (Figure 4A), confirming that the red fluorescence signal originated solely from Rh B. Thus, the fluorescence intensity indirectly reflected the amount of Rh B uptake by the cells. In the experiment involving Rh B-loaded LNPs (composed entirely of EYPC, with a zeta potential of −14 mV), lower uptake was observed in the LO2 cell line, while more significant uptake occurred in the HeLa cell line. This suggests that LNPs preferentially target cancer cells but exhibit low delivery efficiency in normal cells. In contrast, MSLNPs_(EN-βCDs)_ demonstrated high delivery efficiency across both cell types, especially in HeLa cells. Furthermore, the fluorescence intensity per unit area for Rh B-loaded MSLNPs was significantly greater than that of the Rh B-loaded LNP group. This could be attributed to the positive charge and unique architecture of MSLNPs_(EN-βCDs)_, which facilitate easier cellular entry. The results of the pDNA(EGFP) experimental group were consistent with those of the Rh B experimental group (Figure 4B). However, pDNA_(EGFP)_ must be released into the cytoplasm for transcription and translation before green fluorescent protein (GFP protein) can be expressed. The fluorescence intensity per unit area of the LNPs_(SM102)_ group was slightly higher than that of the LNPs group but significantly lower than that of the MSLNPs_(EN-βCDs)_ group, which may be attributed to the weaker positive charge of LNPs_(SM102)_, resulting in inefficient escape from endosomes/lysosomes. The high expression of green fluorescence in MSLNPs_(EN-βCDs)_ indicates their ability to efficiently escape from endosomes/lysosomes after cellular entry and subsequently achieve effective release of pDNA_(EGFP)_.

The intracellular uptake rate of MSLNPs_(EN-βCDs)_ was quantified, as shown in Figure 4C. In the HeLa cell line, the cellular uptake rate of the Rh B drug loaded onto MSLNPs was consistently higher than that of the free Rh B group at all time points. In the LO2 cell line, the cellular uptake rate of the loaded Rh B was significantly higher than that of the free Rh B group in the first 10 h. However, after 10 h, the cellular uptake rate of the loaded Rh B decreased to below that of the free Rh B group. Overall, in the LO2 cell line, the cellular uptake rate of the loaded Rh B group initially increased and then decreased, whereas a continuous upward trend was observed in the HeLa group. The decrease in intracellular Rh B content may be attributable to the preservation of the nanoparticulate structure by MSLNPs_(EN-βCDs)_ within normal cells, followed by their disintegration. In contrast, under the acidic conditions of tumor cells, MSLNPs_(EN-βCDs)_ exhibited efficient release of Rh B, with the Rh B content positively correlated with time. These observations are consistent with the in vitro drug-release profiles of MSLNPs_(EN-βCDs)_.

To further investigate the cellular internalization mechanisms of MSLNPs_(EN-βCDs)_, we conducted cell uptake experiments with Rh B-loaded MSLNPs_(EN-βCDs)_ in the presence of various endocytosis inhibitors. The intracellular content of Rh B was quantitatively measured, and the results of the uptake efficiency are depicted in Figure 4D. Panel (a) illustrates the cellular uptake rates when the caveolae-mediated endocytosis pathway was inhibited by amikacin. The uptake rates of free and loaded Rh B in LO2 and HeLa cell lines were similar to those in the saline group, indicating that neither Rh B nor MSLNPs_(EN-βCDs)_ enter cells via caveolin-mediated pathways. Panel (b) presents the results for the case in which the macropinocytosis pathway was inhibited by cyclosporine A. Compared with the control group, the uptake rate of Rh B in both cell lines remained largely unchanged, while the uptake rate of the MSLNP system was significantly inhibited in both cell lines, with a reduction in over 70% in the HeLa cell line. This suggests that macropinocytosis plays a key role in the internalization of MSLNPs_(EN-βCDs)_. Panel (c) presents the cellular uptake rates when the clathrin-mediated endocytosis pathway was inhibited by chlorpromazine. The uptake of both Rh B and MSLNP were significantly suppressed, with a reduction exceeding 90% in Rh B uptake and a decrease in over 50% in MSLNPs_(EN-βCDs)_ uptake across both cell lines. These results suggest that Rh B was internalized via clathrin-mediated endocytosis, while a portion of MSLNPs_(EN-βCDs)_ also entered the cells through this pathway. These observations illustrate that the intracellular uptake mechanisms of MSLNPs_(EN-βCDs)_ were not limited to a single pathway; instead, multiple endocytic pathways were leveraged to facilitate intracellular delivery.

### 3.10. Biodistribution and Safety Evaluation of MSLNPs_(EN-βCDs)_ in Mice

#### 3.10.1. In Vivo Biodistribution Analysis of MSLNPs_(EN-βCDs)_ in Mice

**Figure 5:**
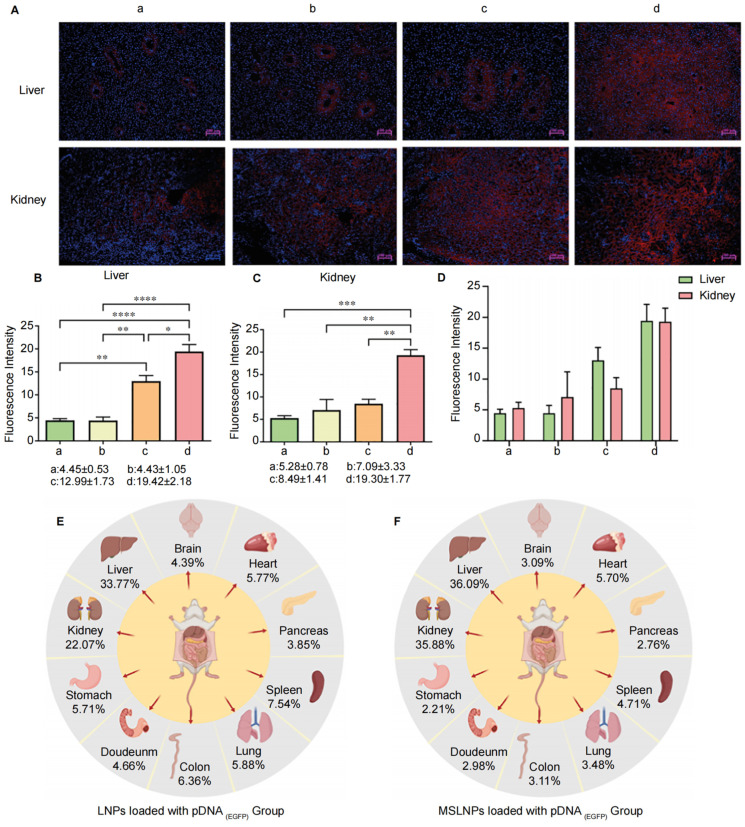
Analysis of MSLNPs distribution in mice. (**A**) Immunofluorescence results of liver and kidney in murine. Quantitative analysis of GFP fluorescence intensity across multiple murine organs: (**B**) liver, (**C**) kidney; (**D**) quantitative analysis of GFP fluorescence intensity across in murine liver and kidney; (**E**,**F**) fluorescence intensity distribution across murine organs in immunofluorescence imaging. (a: Normal saline group. b: pDNA_(EGFP)_ group. c: LNP group. d: MSLNPs group (* *p* < 0.05, ** *p* < 0.01, *** *p* < 0.001, **** *p* < 0.0001)).

The in vivo distribution characteristics of the novel pDNA_(EGFP)_-loaded MSLNP delivery system in major mouse organs (heart, liver, spleen, lungs, kidneys, brain, stomach, pancreas, colon, and duodenum) were systematically evaluated using immunofluorescence imaging. Comparisons were made with control groups, including pDNA_(EGFP)_ loaded onto LNPs, free pDNA_(EGFP)_, and saline. Fluorescence imaging revealed no significant differences in GFP expression between the free pDNA_(EGFP)_ group and negative control group (saline), with both exhibiting only tissue autofluorescence. In contrast, the groups with pDNA_(EGFP)_ loaded onto MSLNPs and LNPs showed distinct biodistribution and GFP expression patterns compared with the controls. The cytoplasmic localization was inferred from the clear separation between the Cy3 signal (indicating GFP expression) and the DAPI (4′,6-diamidino-2-phenylindole)-stained nuclei in high-resolution images (Figure 5A and Figure S13). This finding is consistent with observations in LO2 and HeLa cells.

Further analysis of immunofluorescence intensity across organs (Figure 5B,C and Figure S14) revealed distinct biodistribution patterns. In the LNP group, strong fluorescence signals were observed in the liver and stomach, while other organs exhibited no significant differences compared with the free pDNA_(EGFP)_ and saline control groups. In contrast, the MSLNP group displayed intense fluorescence signals in both the liver and kidneys, with minimal signals in other organs. These results demonstrate that lipid-based nanosystems (MSLNPs_(EN-βCDs)_ and LNPs) enable efficient in vivo delivery of pDNA_(EGFP)_, enhancing expression in the liver and kidneys. Comparative quantification of fluorescence intensity showed that the MSLNP group achieved significantly higher protein expression levels in the liver and kidneys compared with the LNP group (Figure 5E,F).

Notably, renal GFP expression in the MSLNPs_(EN-βCDs)_ group was comparable to hepatic expression, representing a striking deviation from the liver-dominant accumulation characteristic of conventional LNPs. Drawing on our In Vitro cellular uptake and endosomal escape results, we attribute this distinctive biodistribution to the CDs modification, which markedly enhances the nanoparticles’ capacity to evade the reticuloendothelial system (RES) In Vivo. Specifically, the MSLNPs_(EN-βCDs)_ exhibited substantially higher cellular uptake efficiency in both LO2 and HeLa cells, attributable to their positively charged, membrane-mimetic surface architecture. This feature not only facilitates stronger interactions with cellular membranes but also reduces nonspecific adsorption, thereby limiting recognition and clearance by RES phagocytes. More importantly, the robust GFP expression observed following delivery of pDNA_(EGFP)_ by MSLNPs_(EN-βCDs)_ provides direct evidence of efficient endo/lysosomal escape, which is a critical determinant for avoiding the degradative fate typically imposed by RES sequestration. Collectively, these findings demonstrate that CD modification confers dual advantages—enhanced cellular internalization and effective endosomal escape—underpinning the improved RES evasion and the atypical renal accumulation observed. In summary, MSLNPs loaded with pDNA_(EGFP)_ achieved dual hepatic and renal targeting with minimal off-target distribution, distinguishing them from conventional LNPs and highlighting their potential as a versatile platform for modulating biodistribution and improving therapeutic delivery efficiency.

#### 3.10.2. Assessment of MSLNP Immunogenicity

**Figure 6:**
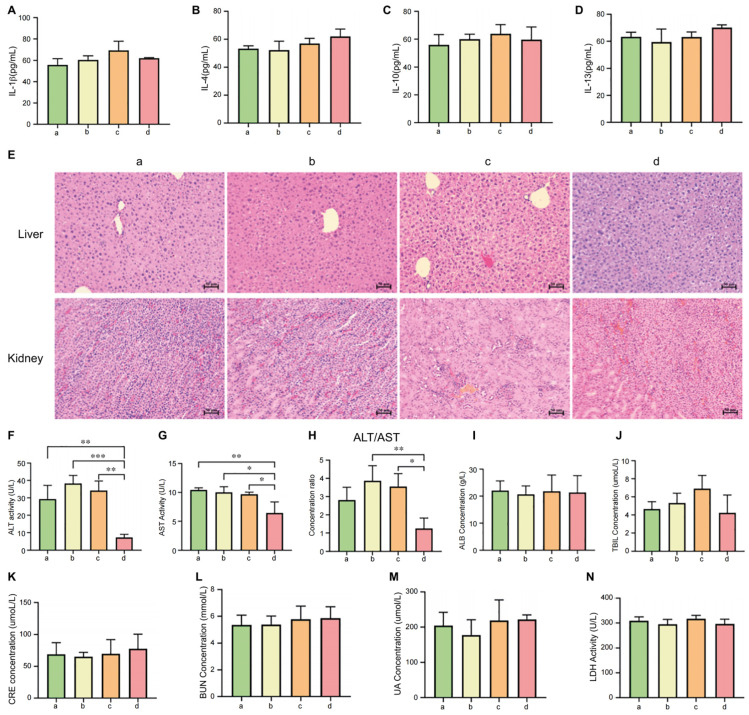
Biosafety evaluation of MSLNPs in mice analysis of expression levels of common inflammatory cytokines ((**A**). IL-1β, (**B**,**C**). IL-4/IL-13, and (**D**). IL-10) in plasma of murine across experimental groups. (**E**) H&E staining of hepatic and renal tissues; ELISA analysis of hepatic and renal function markers in murine across experimental groups: (**F**) AST; (**G**) ALT; (**H**) ALT/AST ratio; (**I**) ALB; (**J**) TBIL; (**K**) CRE; (**L**) BUN; (**M**) UA; (**N**) LDH; (a: normal saline group. b: pDNA_(EGFP)_ group. c: LNP group. d: MSLNP group (* *p* < 0.05, ** *p* < 0.01, *** *p* < 0.001)). IL, interleukin; AST, aspartate aminotransferase; ALT, alanine aminotransferase; ALB, albumin; TBIL, total bilirubin; BUN, blood urea nitrogen; CRE, creatinine; UA, uric acid; LDH, lactate dehydrogenase.

ELISA analysis of plasma inflammatory cytokines (IL-1β, IL-4, IL-10, and IL-13) revealed no significant differences between the MSLNP-based pDNA_(EGFP)_ group and saline control (Figure 6A–D). Comparable IL-1β levels across groups indicated no activation of macrophages, dendritic cells, or the TLR/NF-κB pathway, while uniform IL-4 and IL-13 values ruled out Th2 polarization, humoral immunity, or fibrotic responses. Consistent IL-10 expression further confirmed the absence of immunoregulatory imbalance or myeloid-derived suppressor cell activation. Notably, MSLNPs_(EN-βCDs)_ exhibited markedly lower immunogenicity than conventional cationic liposomes. This can be attributed to the excellent biocompatibility of their components (amino-β-CD, linoleic acid, and EYPC) and their efficient acidic pH-responsive endosomal escape, which avoids prolonged lysosomal retention and subsequent inflammasome activation.

#### 3.10.3. Assessment of Hepatic and Renal Functions

In nanomedicine and drug delivery system research, assessing liver and kidney function is critical for evaluating the safety and clinical translation potential of novel carriers. To further investigate the biocompatibility, potential toxicity, and In Vivo metabolic mechanisms of MSLNPs_(EN-βCDs)_, we analyzed the histological structure and functional biochemical markers of the liver and kidneys in mouse models. Physiological parameters across experimental groups were measured, with results summarized in Figure 6.

Hematoxylin and eosin (H&E) staining results of hepatic and renal tissues are shown in Figure 6E. Tissue sections from all experimental groups showed normal histoarchitecture, characterized by regularly shaped (round or oval) dark blue nuclei, uniformly distributed eosinophilic cytoplasm, and an intact extracellular matrix. No evidence of inflammatory cell infiltration (e.g., neutrophils or lymphocytes) was found. These findings indicate that none of the experimental treatments induced significant histomorphological alterations in hepatic or renal tissues during the experimental period.

The liver, as the primary metabolic organ for most drugs, reflects drug-induced toxicity through key biochemical markers. Alanine aminotransferase (ALT, specific marker for hepatocyte injury), aspartate aminotransferase (AST, indicative of hepatic/muscle damage), albumin (ALB, reflecting hepatic synthetic function and nutritional status), and total bilirubin (TBIL, assessing biliary excretion and hemolytic disorders) were analyzed. As shown in Figure 6F–H, the MSLNPs group exhibited significantly lower ALT and AST levels compared with the LNP and free pDNA_(EGFP)_ groups, with ALT/AST ratios similar to those for the saline control. In contrast, the LNP and free pDNA groups showed slightly elevated ALT/AST ratios (less than twofold increase) and marginally higher TBIL levels, suggesting mild hepatic stress. However, no significant differences in ALB or TBIL were observed across groups, indicating preserved hepatic synthetic function and nutritional status at the tested dosage.

The kidneys, critical for drug excretion, were evaluated via renal function markers: blood urea nitrogen (BUN, indicating glomerular filtration rate and renal blood flow), creatinine (CRE, reflecting glomerular filtration rate), and uric acid (UA, associated with purine metabolism and tubular reabsorption). Figure 6K–M demonstrate no intergroup differences in CRE, BUN, UA, or BUN/CRE ratios among the MSLNP, LNP, free pDNA, and saline groups, confirming no renal toxicity. Additionally, plasma lactate dehydrogenase (LDH) levels, a broad indicator of cellular damage, showed no significant variations across groups (Figure 6N), consistent with normal hepatic (ALT/AST) and renal (CRE) parameters, collectively indicating systemic biocompatibility.

In summary, the comprehensive analysis of hepatic and renal function markers, combined with low immunogenicity, demonstrates the high biosafety profile of MSLNPs_(EN-βCDs)_ for In Vivo drug delivery.

## 4. Conclusions

We constructed kidney-targeted, acidic pH-responsive MSLNPs, primarily based on amphiphilic HGLs derived from amine β-CDs. In the HGL, amine-modified CDs provided suitable positive charge and pH responsiveness, while the single-chain linoleic acid offered necessary structural support and a low molecular weight, facilitating the assembly of MSLNPs. Through structure–activity relationship studies, we found that the addition of EYPC substantially improved the stability of MSLNPs. Following rigorous evaluation of stability under diverse testing conditions and MD simulations, the optimal MSLNP formulation was identified, and its delivery performance and mechanistic behavior were comprehensively assessed. MSLNPs demonstrated high encapsulation efficiency for both hydrophilic and hydrophobic drugs. In vitro drug-release experiments and studies on delivery performance in biological systems indicated that MSLNPs exhibit acidic pH-responsive properties, capable of rapid drug release in slightly acidic environments while maintaining stability in neutral environments. Additionally, biological data showed that MSLNPs can be efficiently internalized by various types of cells through multiple pathways. Notably, MSLNPs exhibited preferential renal-targeting capability in murine models, distinct from the liver-targeting specificity observed in conventional LNPs, while demonstrating favorable biosafety profiles with low immunogenicity. The successful construction of MSLNPs provides a promising strategy for expanding lipid libraries and simplifying LNP formulations. The successful construction of MSLNPs provides alternative strategies and reference points for the lipid library. Due to experimental conditions and time constraints, several areas remain open for future investigation. For example, it is unclear whether microfluidic parameters influence the encapsulation efficiency and other physicochemical properties of MSLNPs. It is also uncertain whether varying the type of amino cyclodextrin could help identify suitable auxiliary lipid molecules for achieving similarly efficient delivery and whether the structure–activity relationship corresponds with that of MSLNPs. In addition, further refinement of the HGL-to-EYPC ratio may potentially enhance the formulations. However, all these questions necessitate more in-depth investigation.

## Figures and Tables

**Table 1:** Physicochemical properties of pDNA_(EGFP)_ in LNPs, MSLNPs_(EN-βCDs)_, and LNPs_(SM102)_ formulations.

Experimental Conditions	Encapsulation Efficiency (%)	Drug-Loading Capacity (ng/mL)	Particle Size (nm)	Zeta Potential (mV)	Maximum PDI
MSLNPs_(EN-βCDs)_ loaded with pDNA_(EGFP)_	91.25	99.0	142.4 ± 0.8	11.57 ± 0.39	0.092
LNPs loaded with pDNA_(EGFP)_	35.32	23.3	181.9 ± 2.0	−5.14 ± 0.75	0.210
LNPs_(SM102)_ loaded with pDNA_(EGFP)_	88.25	78.7	167.9 ± 1.1	5.34 ± 0.25	0.112

## Data Availability

All data generated or analyzed during this study are included in this published article (and its Supplementary Information files).

## Supplementary Materials

The following supporting information can be downloaded at https://www.mdpi.com/article/10.3390/pharmaceutics17111410/s1, Figure S1: (A) 1H NMR and (B) HR-MS of DMEN-βCDs; Figure S2: (A) 1H NMR and (B) HR-MS of DEEN-βCDs; Figure S3: 1H NMR and (B) HR-MS of AEP-βCDs; Figure S4: 1H NMR and (B) HR-MS of AEMO-βCDs; Figure S5: (A) 1H NMR and (B) HR-MS of linoleic acid–Ad; Figure S6: pKa data for eight types of amine β-CDs and their HGLs; Figure S7:Titration NMR spectra of EN-βCDs and linoleic acid-Ad at different concentrations. From bottom to top, EN-βCDs, linoleic acid–Ad, 10 mg EN-βCDs, 12 mg EN-βCDs, 14 mg EN-βCDs, 16 mg EN-βCDs, and 20 mg EN-βCDs; Figure S8: Box plots of particle size measurements for MSLNPs constructed with 100% HGL_EN-βCDs_ under different conditions; Figure S9: Self-assembly molecular dynamics simulations of MSLNPs(DETA-βCDs); Figure S10: UV standard curve of Rh B, Abs = 103831.15113(Concentration of Rh B)–0.08053, R2 = 0.9992; Figure S11: UV standard curve of CBD, Abs = 994.46078(Concentration of CBD)–0.01061, R2 = 0.9986; Figure S12: Sequence structure of pcDNA3.1-EGFP; Figure S13: Immunofluorescence results of heart, spleen, lungs, brain, stomach, pancreas, colon, and duodenum in murine; Figure S14:Quantitative analysis of GFP fluorescence intensity across multiple murine organs. Table S1: EN-βCDs and linoleic acid–Ad NMR titration sample concentrations and peak area data; Table S2: MSLNP particle size, zeta potential, and DPI data associated with different HGL_(EN-βCDs)_/EYPC ratios; Table S3: MSLNP particle size, zeta potential, and DPI data associated with different HGL_(DETA-βCDs)_/EYPC ratios; Table S4: MSLNP particle size, zeta potential, and DPI data associated with different HGL_(TETA-βCDs)_/EYPC ratios; Table S5: MSLNP particle size, zeta potential, and DPI data associated with different HGL_(DMEN-βCDs)_/EYPC ratios; Table S6: MSLNP particle size, zeta potential, and DPI data associated with different HGL(DEEN-βCDs)/EYPC ratios; Table S7: MSLNP particle size, zeta potential, and DPI data associated with different HGL(AEP-βCDs)/EYPC ratios; Table S8: MSLNP particle size, zeta potential, and DPI data associated with different HGL(AEMO-βCDs)/EYPC ratios; Table S9: MSLNP particle sizes associated with different HGL/EYPC ratios, and zeta potential at different temperatures and time (PDI < 0.20 for all groups); Table S10: Encapsulation efficiency and drug loading capacity of Rh B and CBD in LNPs, MSLNPs(EN-βCDs), and LNPs(SM102) formulations.

## Abbreviations

The following abbreviations are used in this manuscript:

LNPs	lipid nanoparticles
β-CD	β-cyclodextrin
HGL	host–guest amphiphilic lipid molecules
MSLNPs	multi-stage assembly supramolecular LNPs
