# Supplementary material for: Supramolecular Lipid Nanoparticles Based on Amine β-CD Host–Guest Lipids: Design, Mechanisms, and Biosafety
Source: Pharmaceutics. 2025 Oct 30;17(11):1410. doi: 10.3390/pharmaceutics17111410 (PMC12655198; doi:10.3390/pharmaceutics17111410)
Supplement: Supplementary file 1 [file pharmaceutics-17-01410-s001.zip › pharmaceutics-3878118-supplementary.pdf]

Supplementary Information

Lv et. al.

Supramolecular Lipid Nanoparticles Based on Amine β-CD Host–Guest Lipids: Design, Mechanisms, and Biosafety

Pin Lv[1, 2, 3*]

Yamin Li[1*]

Gang Du [1*]

Jiawei Ding[1*]

Jiawei Zhou[1*]

Yuan Zhang[2, 3]

Huang Lin[2, 3]

Ming Yang[2, 3]

Chao Zhou[1]

Bo Yang[1*]

[1]Faculty of Life Science and Technology, Kunming University of Science and Technology, Kunming 650500, P. R. China; [2]Industrial Crop Research Institute, Yunnan Academy of Agricultural Sciences, Kunming 650500, P. R. China; [3]Yunnan Key Laboratory of Genetic Improvement of Herbal Oil Crops, Kunming 650500, P. R. China

*These authors contributed equally to this work

Correspondence: Bo Yang

Faculty of Life Science and Technology

Kunming University of Science and Technology

Kunming 650500

P. R. China

Tel: +86 871 5920570

Fax: +86 871 5920570

E-mail: yangbo6910@sina.com

Synthesis of Amine β-CDs and Linoleic acid–Ad

6-OTs-βCD

A total of 0.42 kg (0.38 mol) of β-CD was dissolved in 2.6 L of water. After stirring thoroughly, a solution of NaOH (34.4 g, 8.6 M) was slowly added. The mixture was allowed to react for 2 h following the complete addition of the NaOH solution. TsCl (0.28 mol) dissolved in 160 mL of acetonitrile was then gradually added, and the reaction mixture was stirred for an additional 2 h. Upon completion of the reaction, the mixture was vacuum filtered to collect the filtrate. The filtrate was adjusted to pH = 7.5 using 2 M of hydrochloric acid solution. After standing for 24 h, the mixture was again vacuum filtered to obtain a white solid. This white solid was dissolved in 900 mL of boiling water for recrystallization, and the filtrate was collected. After three rounds of recrystallization, the white precipitate was collected and vacuum dried for 48 h to yield 29.9 g of 6-OTs-βCD, with an approximate yield of 7.4%.

Electrospray ionization mass spectrometry data (ESI-MS): *m*/*z* 1288.38. ^{1}H NMR (600 MHz, DMSO-d_6): δ 2.39 (s, 3H, CH_3), 3.19–3.59 (m, 40H, H-2, 4, 6 of CD, and H-3, 5 of CD), 4.29–4.50 (m, OH), 4.90 (s, 7H, H-1 of CD), 5.65–5.90 (m, OH), 7.43 (d, 2H, Ar-H), 7.76 (d, 2H, Ar-H).

N_3-βCD

A total of 2.52 g of 6-OTs-βCD and 0.60 g of NaN_3 were added to 10 mL of DMF. The mixture was ultrasonically stirred until complete dissolution and allowed to react under a nitrogen atmosphere at 75 °C for 10 h. Thin-layer chromatography (TLC) was used to monitor the reaction progress, with the eluent composed of isopropanol, ethyl acetate, aqueous ammonia, and water

in a ratio of 7:7:5:4. After the reaction of 6-OTs-βCD was complete, the reaction mixture was slowly added to 100 mL of acetone. The mixture was stirred for 0.5 h, and the solids were then collected by filtration. The solids were then washed twice with acetone and collected again by filtration. Vacuum drying yielded 2.4 g of N_3-βCD, with a yield of 79%.

ESI-MS: *m*/*z* 1159.38. ^{1}H NMR (600 MHz, D_2O): δ 3.40–3.79 (m, H-2, 3, 4, 5, 6 of CD), 4.89 (s, 7H, H-1 of CD).

NH_2-βCD

A total of 6.0 g of N_3-βCD and 2.85 g of triphenylphosphine (PPh_3) were added to 30 mL of DMF. The mixture was allowed to react at room temperature for 4 h, with TLC used to monitor the reaction progress. After the reaction was completed, 20 mL of aqueous ammonia was added dropwise, and the reaction continued at room temperature for an additional 18 h. The purification was performed using a solvent–antisolvent method, leveraging the distinct solubility profiles of the reactants: the amine-βCDs exhibit good water solubility but are insoluble in acetone, while the other starting materials show certain solubility in acetone. A water-to-acetone volume ratio of 1:10 was maintained during the purification process. The reaction mixture was then filtered, and the filtrate was slowly added to 200 mL of acetone and stirred for half an hour before collecting the solids by filtration. Vacuum drying yielded 5.89 g of NH_2-βCD, with a yield of 82%. Purity: 92.1% by HPLC.

ESI-MS: *m*/*z* 1133.38. ^{1}H NMR (600 MHz, D_2O) δ 3.32–3.96 (m, H-2, 3, 4, 5, 6 of CD), 5.01 (s, 7H, H-1 of CD).

EN-βCD

A total of 6 g of 6-OTs-βCD was added to 30 mL of ethylenediamine solution. Under a nitrogen atmosphere, the reaction was carried out at 80 °C for 20 h. After the reaction, the mixture was added to 100 mL of acetone to precipitate the solid. The solid was then dissolved in 10 mL of ultrapure water and reprecipitated by adding the solution to 100 mL of acetone. This process was

repeated three times. Vacuum drying yielded 3.42 g of EN-βCD, with a yield of 78%. Purity: 94.0% by HPLC.

ESI-MS: *m*/*z* 1176.42. ^{1}H NMR (600 MHz, D_2O) δ 2.59–2.88 (m, 4H, –CH_2CH_2– of ethylenediamine), 3.40–3.79 (m, H-2, 3, 4, 5, 6 of CD), 5.00 (s, 7H, H-1 of CD).

DETA-βCD

A total of 6 g of 6-OTs-βCD was added to 30 mL of diethylenetriamine solution. Under a nitrogen atmosphere, the reaction was carried out at 80 °C for 20 h. After the reaction, the mixture was added to 100 mL of acetone to precipitate the solid, which was retained. The solid was then washed three times with 50 mL of acetone. Vacuum drying yielded 3.87 g of DETA-βCD, with a yield of 60%. Purity: 91.9% by HPLC.

ESI-MS: *m*/*z* 1219.47. ^{1}H NMR (600 MHz, D_2O) δ 2.59–2.88 (m, 8H, –CH_2CH_2– of diethylenetriamine), 3.40–3.79 (m, H-2, 3, 4, 5, 6 of CD), 5.00 (s, 7H, H-1 of CD).

TETA-βCD

A total of 6 g of 6-OTs-βCD was added to 44 mL of triethylenetetramine solution. Under a nitrogen atmosphere, the reaction was carried out at 80 °C for 20 h. After the reaction, the mixture was added to 100 mL of acetone to precipitate the solid, which was retained. The solid was then washed three times with 50 mL of acetone. Vacuum drying yielded 4.16 g of TETA-βCD, with a yield of 50%. Purity: 93.6% by HPLC.

ESI-MS: *m*/*z* 1262.51. ^{1}H NMR (600 MHz, D_2O) δ 2.59–2.88 (m, 12H, –CH_2CH_2– of triethylenetetraamine), 3.40–3.79 (m, H-2, 3, 4, 5, 6 of CD), 5.00 (s, 7H, H-1 of CD).

DMEN-βCD

A total of 6 g of 6-OTs-βCD was dissolved in 30 mL of dimethylaminoethylamine solution. Under a nitrogen atmosphere, the reaction was carried out at 70 °C for 20 h. After cooling to room temperature, the reaction mixture was added to 100 mL of acetone to precipitate the solid, which

was retained. The solid was then washed three times with 200 mL of acetone. Vacuum drying yielded 3.96 g of DMEN-βCD, with a yield of 65%. Purity: 93.7% by HPLC.

ESI-MS: *m*/*z* 1205.4668. ^{1}H NMR (600 MHz, D_2O) δ 2.15–2.25 (s, 6H, $–CH_3$ of dimethylaminoethylamine), 2.59–2.88 (m, 12H, $–CH_2CH_2–$ of dimethylaminoethylamine), 3.40–3.79 (m, H-2, 3, 4, 5, 6 of CD), 5.00 (s, 7H, H-1 of CD).

DEEN-βCD

A total of 6 g of 6-OTs-βCD was weighed and dissolved in 30 mL of diethylethylenediamine solution. Under a nitrogen atmosphere, the reaction was carried out at 70 °C for 20 h. After cooling to 20–25 °C, the reaction mixture was added to 100 mL of acetone to precipitate the solid, which was retained. The solid was then washed three times with 200 mL of acetone. Vacuum drying yielded 4.15 g of DEEN-βCD, with a yield of 70%. Purity: 90.9% by HPLC.

ESI-MS: *m*/*z* 1233.4978. ^{1}H NMR (600 MHz, D_2O) δ 2.15–2.25 (s, 6H, $–CH_3$ of dimethylaminoethylamine), 2.59–2.88 (m, 12H, $–CH_2CH_2–$ of dimethylaminoethylamine), 3.40–3.79 (m, H-2, 3, 4, 5, 6 of CD), 5.00 (s, 7H, H-1 of CD).

APE-βCD

A total of 6 g of 6-OTs-βCD and 1 g of 1-(2-aminoethyl)pyrrolidine were dissolved in 30 mL of DMF. Under a nitrogen atmosphere, the reaction was carried out at 70 °C for 20 h. After cooling to room temperature, the reaction mixture was added to 100 mL of acetone to precipitate the solid, which was retained. The solid was then washed three times with 200 mL of acetone. Vacuum drying yielded 4.15 g of AEP-βCD, with a yield of 66%. Purity: 91.8% by HPLC.

ESI-MS: *m*/*z* 1231.4820. ^{1}H NMR (600 MHz, D_2O) δ 1.85 (s, 4H, $–CH_2–$ of 1-(2-aminoethyl)pyrrolidine), 2.6–2.93 (m, $–CH_2–$ of 1-(2-aminoethyl)pyrrolidine), 3.00–3.79 (m, H-2, 3, 4, 5, 6 of CD), 5.00 (s, 7H, H-1 of CD).

AEMO-βCD

A total of 6 g of 6-OTs-βCD and 1.3 g of 4-(2-aminoethyl)morpholine were dissolved in 30 mL of DMF. Under a nitrogen atmosphere, the reaction was carried out at 80 °C for 20 h. After cooling to room temperature, the reaction mixture was added to 100 mL of acetone to precipitate the solid, which was retained. The solid was then washed three times with 200 mL of acetone. Vacuum drying yielded 4.27 g of AEMO-βCD, with a yield of 59%. Purity: 95.2% by HPLC.

ESI-MS: *m*/*z* 1247.4774. ^{1}H NMR (600 MHz, D_2O) δ 2.4–2.75 (s, 8H, $–CH_2–$ of 4-(2-aminoethyl)morpholine), 2.6–2.8 (m, $–CH_2–$ of 4-(2-aminoethyl)morpholine), 3.00–4.00 (m, H-2, 3, 4, 5, 6 of CD and H-10 of 4-(2-aminoethyl)morpholine), 5.00 (s, 7H, H-1 of CD).

Linoleic Acid–Ad

A total of 280 mg (1 mmol) of linoleic acid was dissolved in 15 mL of dichloromethane. The solution was stirred at 0 °C for 10 min. Subsequently, EDCI (230 mg, 1.2 mmol) and HOBT (162 mg, 1.2 mmol) were added to activate the mixture under stirring for 1 h. Next, adamantylamine (302.5 mg, 2 mmol) was introduced into the reaction mixture. The reaction proceeded under nitrogen atmosphere at room temperature for 24 h. Reaction progress was monitored by TLC using a solvent system of petroleum ether/ethyl acetate in a 3:1 ratio. Purification via column chromatography yielded a colorless oil characterized as linoleic acid–Ad, with a mass of 517 mg and a yield of 93%. Purity: 97.5% by HPLC.

ESI-MS: *m*/*z* 414.3730. ^{1}H NMR (600 MHz, $CDCl_3$) δ 0.75 (s, 3H, H-1), 1.20–1.40 (m, 16H, H-2, 3, 4, 12, 13, 14, 15, 16), 1.55–1.75 (m, 9H, H of amantadine), 1.90–1.95(m, 6H, H of amantadine), 2.06 (m, 6H, H-5, 11, 17), 2.77 (m, 2H, H-8), 5.12(s, H of –NH-), 5.30–5.42(m, 4H, H-6, 7, 9, 10).

General Methods

Determination of pKa Values

Amine β-CDs (or linoleic acid–Ad/amine β-CDs inclusion complex) aqueous solutions were prepared with a concentration of 1.5 mg/mL. Titrations were performed by adding 0.1 M hydrochloric acid (3, 6, or 12 μL), and the resulting pH values were recorded. A titration curve was plotted with the pH value as the ordinate and the total volume of added hydrochloric acid as the abscissa. Subsequently, inflection points were identified, indicating the endpoint of titration. The pH value corresponding to this inflection point was considered the pKa value.

Phase-Solubility Diagram

The phase–solubility diagram was obtained following the method of Higuchi and Connors. For the ^{1}H NMR analysis, a solvent mixture of D_2O and CH_3OH in a ratio of 8:2 with a total volume of 1 mL was selected. The concentration of linoleic acid–Ad was fixed at 2.417×10^{-2} M, equivalent to 10 mg/mL. Subsequently, amine β-CDs are added to this solution in increasing molar amounts: 10 mg (0.3514 equivalent), 12 mg (0.4217 equivalent), 14 mg (0.4920 equivalent), 16 mg (0.5623 equivalent), and 20 mg (0.7029 equivalent), followed by stirring in the dark for 72 h. The solutions were filtered to remove insoluble matter and then filtered again using a 0.45 μm microporous membrane. This process was repeated three times. NMR was used to detect the ratio of linoleic acid–Ad to the corresponding CD peak areas, from which the concentration of linoleic acid–Ad was calculated. The solubility phase diagram was then constructed by plotting the CD concentration against the linoleic acid–Ad concentration.

SEM Analysis

SEM analysis was conducted using a JEOL JSM 840 scanning electron microscope (JEOL Ltd., Japan). The samples were mounted on metal stubs with double-sided adhesive tape. To avoid

electrical charging owing to sample insulation, a thin layer of gold was sputtered on top of the samples prior to the SEM scan.

Particle Size and Zeta Potential Measurements

DLS was applied to determine the particle size and zeta potential of the sample using a Zetasizer-Nano ZS90 instrument (Malvern Instruments Ltd., Worcestershire, U.K.). The total concentration of lipid molecules was controlled at 100 μM.

TEM Experiments

TEM images were captured using a Tecnai G2 F30 S-TWIN microscope operating at an accelerated voltage of 200 kV. Samples were prepared by placing a few drops of the solution onto a copper grid, followed by air drying the grid to remove any excess solvent prior to imaging.

^{1}H NMR and 2D-ROESY NMR

^{1}H NMR and 2D ROESY NMR spectra were acquired on a Bruker Avance DRX spectrometer operated at 600 MHz and 298 K. All NMR experiments were conducted in D_2O or DMSO. Tetramethylsilane (TMS) was used as a reference. Samples were dissolved in 99.98% D_2O and filtered before use.

Encapsulation Efficiency and Drug Loading Capacity

The precise measurement of Rh B (or CBD) was first conducted, followed by the preparation of MSLNPs loaded with Rh B (or CBD). The reaction solution was dialyzed (MWCO = 5 KDa, time = 6 h), and the absorbance of the dialysis solution at 540 nm (or 254 nm) was measured. The Rh B (or CBD) content was calculated by substituting into the standard curve, and the results were used to compute the encapsulation efficiency according to the formula provided below,

$$Encapsulation\ Efficiency(\%) = \frac{(M_2 - M_1)}{M_2} \times 100\%,$$

where M_1 is the content of Rh B (or CBD) in the dialysis solution, and M_2 is the initial mass of Rh B (or CBD). To ensure accuracy, each measurement was repeated three times.

The MSLNPs solution loaded with Rh B (or CBD) was lyophilized, accurately weighed, and subjected to thorough demulsification. The content of Rh B (or CBD) was then detected using HPLC, and the drug loading capacity was calculated using the following formula:

$$Drug\ Loading\ Capacity(\%) = \frac{M_3}{M_4} \times 100\%,$$

where M_3 is the content of Rh B (or CBD) in the dialysis solution, and M_4 is the initial mass of MSLNPs. To ensure accuracy, each measurement was repeated three times.

Drug Release

MSLNPs loaded with Rh B (containing 400.0 mmol of HGL and 400.0 mmol of EYPC), with an encapsulation efficiency of 33.99%, were prepared in 800 mL of different solutions at 37 °C (mimic the core body temperature): Tris buffer (pH = 7.4), citrate buffer (pH = 6.0), hydrochloric acid buffer (pH = 4.5), Dulbecco's Modified Eagle Medium supplemented with fetal bovine serum (FBS, 10%), and saline. Each 800 mL solution was divided into eight equal parts, and samples of 1 mL were extracted at 0.5, 1, 2, 6, 12, 24, 48, and 72 h. These samples were centrifuged at 10000 r/min for 15 min, and the supernatant was collected for high-performance liquid chromatography (HPLC) to determine the Rh B content. To ensure accuracy, each measurement was repeated three times. The release efficiency was determined using the following,

$$Release\ Efficiency(\%) = \frac{100Q_1}{\frac{Q_2}{8}} \times 100\%,$$

where Q_1 is the detected content of Rh B, and Q_2 is the initial weighed mass of Rh B.

Demulsification Experiment

Dialyzed MSLNPs loaded with Rh B (containing 15 mmol of HGL and 15 mmol of EYPC), with an encapsulation efficiency of 36.7%, were dispersed in 30 mL of Tris buffer for subsequent use. A 1 mL aliquot of this solution was placed into a 25 mL volumetric flask, and a demulsifier (Triton X-100 or methanol or a methanol/chloroform mixture) was added. After thorough mixing, the solution was sonicated for 5 min, followed by centrifugation at 10000 r/min for 15 min. The

supernatant was collected and analyzed using HPLC, and the demulsification rate was calculated using the following,

$$Demulsification\ rate(\%) = (D_1 \times D_2) \times 100\%,$$

where D_1 is the Rh B content in the supernatant after demulsification, and D_2 is the initial Rh B content. To ensure accuracy, each measurement was repeated three times.

Cellular Uptake Assays

Rh B (or $pDNA_{(EGFP)}$) was used as an indicator in uptake experiments with cells (LO2 and HeLa), and consistent amounts were applied across all groups. Cells were cultured in complete DMEM supplemented with FBS (10%) at 37 °C in a CO_2 (5%) atmosphere with a relative humidity of 95%. Cells were seeded in 24-well plates at a density of 5×10^4 cells per well. After 48 h (internalization inhibitors added at the 24 h mark: chlorpromazine 5 mM, cyclosporine A 3 nM, or amikacin 1 μM), the culture medium was replaced with a medium containing 10% FBS and MSLNPs loaded with Rh B (10 μg/mL). The encapsulation efficiency was 25%. After incubating the cells with MSLNPs for 1, 2, 4, 6, 8, 10, 12, and 24 h (internalization inhibition experiments conducted at the 24-hour mark), the original culture medium was replaced with fresh culture medium containing 10% FBS (500 μL), and the cells were incubated under the same conditions for an additional 12 h. After incubation, the samples were split into two groups: one for CLSM observation, and the other digested with trypsin, followed by centrifugation to collect the supernatant, which was subjected to HPLC for concentration quantification. The uptake rate was calculated using the following,

$$\text{Uptake rate (\%)} = \frac{\text{Content of Rh B in the supernatant}}{\text{Initial content of Rh B in the culture medium}} \times 100\%,$$

and the initial content of Rh B in the culture medium was 10 μg/mL.

In Vivo Administration of MSLNPs in Mice

A total of 24 experimental mice were randomly divided into four groups: Group A (Negative control): 100 μL saline; Group B ($pDNA_{(EGFP)}$-treated): 100 μL $pDNA_{(EGFP)}$ plasmid solution (300

ng, 11 μL plasmid + 89 μL PBS); Group C (LNPs loaded with $pDNA_{(EGFP)}$): 100 μL LNPs (plasmid concentration of 300 ng/mL); Group D (MSLNPs loaded with $pDNA_{(EGFP)}$): 100 μL MSLNPs (plasmid concentration of 300 ng/mL). Following tail vein injection (at the 12-hour time point), major organs (heart, liver, spleen, lungs, kidneys, brain, stomach, pancreas, small intestine, and large intestine) were harvested. Half of each organ processed into paraffin sections. Organ sections were sequentially incubated with GFP primary antibody and Cy3-conjugated secondary antibody, as well as counterstained with DAPI. This was followed by fluorescence microscopy imaging. GFP protein expression levels in organs were quantified using ELISA kits. Serum immune factors and hepatic/renal function markers were analyzed. All mice were maintained under identical housing conditions to ensure experimental comparability and data reliability. Additionally, H&E staining was performed using hematoxylin and eosin solutions as the staining agents. The procedural steps (e.g., tissue sectioning, dewaxing, and mounting) were similar to those in immunofluorescence protocols, although the core staining mechanisms were different.

Table S1 EN-βCDs and linoleic acid–Ad NMR titration sample concentrations and peak area data.

Experimental Conditions		1	2	3	4	5
EN-βCDs	Concentration (Mm)	8.5×10^{-3}	1.02×10^{-2}	1.19×10^{-2}	1.36×10^{-2}	1.7×10^{-2}
	Characteristic peak area (H-1 of EN-βCDs)	7	7	7	7	7
linoleic acid-Ad	Concentration (mM)	2.12×10^{-5}	5.1×10^{-5}	7.44×10^{-5}	1.1×10^{-4}	1.70×10^{-4}
	Characteristic peak area (H-2, 3, 4, 12, 13, 14, 15,	0.04	0.08	0.11	0.13	0.16

	16 of linoleic acid–Ad)					

Table S2 MSLNP particle size, zeta potential, and DPI data associated with different $HGL_{(EN\text{-}\beta CDs)}$/EYPC ratios

HGL (EN-βCDs) (mol%)	EYPC (mol%)	Undiluted			Tenfold diluted		
		Particle size (nm)	Zeta potential (mV)	Maximum PDI	Particle size (nm)	Zeta potential (mV)	Maximum PDI
90	10	296.7±17.1	+43.85±2.34	0.445	1869.6±89.4	–0.01±1.03	1.00
80	20	273.9±9.3	+37.28±0.92	0.351	984.5±53.2	+3.47±1.98	0.817
70	30	222.4±3.0	+29.46±0.84	0.178	406.8±20.6	+22.73±0.87	0.485
60	40	150.2±2.7	+18.69±0.63	0.166	157.0±5.7	+17.87±0.51	0.182
50	50	142.3±1.1	+13.23±0.27	0.093	142.5±2.1	+13.31±0.34	0.099
40	60	144.1±1.3	+9.17±0.36	0.097	145.7±1.8	+10.24±1.01	0.098
30	70	150.6±1.9	+4.28±0.09	0.126	179.3±3.5	+3.61±0.65	0.101
20	80	155.2±1.4	–0.07±0.03	0.144	184.1±2.9	–1.98±0.72	0.151
10	90	169.7±2.0	–2.34±0.19	0.163	201.8±1.8	–2.69±0.09	0.205
0	100	180.3±2.2	–5.31±0.23	0.182	260.7±4.1	–5.24±0.34	0.180

Table S3 MSLNP particle size, zeta potential, and DPI data associated with different $HGL_{(DETA\text{-}\beta CDs)}$/EYPC ratios

HGL (DETA-βCDs) (mol%)	EYPC (mol%)	Undiluted			Tenfold diluted		
		Particle size (nm)	Zeta potential (mV)	Maximum PDI	Particle size (nm)	Zeta potential (mV)	Maximum PDI

90	10	294.0±18.8	+51.17±4.25	0.622	2050.4±103.4	–1.84±0.93	1.00
80	20	286.7±11.1	+43.42±4.25	0.385	1020.3±58.7	+4.26±2.35	0.856
70	30	234.2±5.2	+37.82±1.33	0.216	630.9±22.9	+25.98±1.27	0.732
60	40	219.1±5.6	+29.66±1.15	0.187	369.3±6.4	+19.23±0.74	0.318
50	50	188.6±3.9	+20.47±0.81	0.112	223.8±2.6	+14.91±0.59	0.122
40	60	152.6±3.1	+14.74±0.44	0.096	158.4±2.1	+11.75±1.33	0.107
30	70	175.2±2.5	+10.27±0.50	0.141	192.7±4.1	+4.81±0.94	0.116
20	80	186.4±1.9	+4.61±0.22	0.164	208.5±3.3	–2.37±0.85	0.171
10	90	187.9±2.4	–1.21±0.36	0.197	238.6±2.4	–3.56±0.28	0.234
0	100	183.7±3.1	–5.28±0.41	0.201	264.1±3.7	–5.04±0.21	0.184

Table S4 MSLNP particle size, zeta potential, and DPI data associated with different HGL$_{(TETA-\beta CDs)}$/EYPC ratios

HGL $_{(TETA-\beta CDs)}$ (mol%)	EYPC (mol%)	Undiluted			Tenfold diluted		
		Particle size (nm)	Zeta potential (mV)	Maximum PDI	Particle size (nm)	Zeta potential (mV)	Maximum PDI
90	10	318.7±21.5	+55.12±5.31	0.659	2251.1±115.8	–3.24±1.03	1.00
80	20	301.4±11.2	+48.63±3.65	0.418	1075.1±62.4	–3.47±1.98	0.995
70	30	281.8±4.8	+39.19±1.47	0.353	585.3±24.7	+1.17±0.75	0.898
60	40	248.5±3.9	+30.92±1.99	0.445	375.6±17.1	+21.75±0.96	0.614
50	50	205.0±1.8	+24.45±1.06	0.351	275.4±10.4	+18.19±0.67	0.471
40	60	174.2±2.1	+19.56±0.87	0.217	204.1±6.5	+17.87±1.43	0.315
30	70	157.6±2.7	+15.97±0.49	0.098	186.2±3.0	+13.23±0.52	0.112
20	80	176.3±2.3	+6.59±0.98	0.192	232.8±3.8	–3.17±0.97	0.314

10	90	190.4±3.0	–4.22±1.41	0.228	250.5±8.9	–5.64±1.33	0.457
0	100	188.9±3.6	-5.18±0.54	0.194	259.9±5.7	–6.01±0.43	0.197

Table S5 MSLNP particle size, zeta potential, and DPI data associated with different $HGL_{(DMEN\text{-}\beta CDs)}$/EYPC ratios

HGL (DMEN-βCDs) (mol%)	EYPC (mol%)	Undiluted			Tenfold diluted		
		Particle size (nm)	Zeta potential (mV)	Maximum PDI	Particle size (nm)	Zeta potential (mV)	Maximum PDI
90	10	242.4±19.5	+9.45±4.81	0.689	1980.6±108.2	–3.91±1.34	1.00
80	20	202.7±2.7	+4.76±3.90	0.442	1258.2±63.8	–2.35±2.11	0.912
70	30	188.9±5.6	–0.35±0.09	0.280	598.3±26.9	–3.02±1.76	0.786
60	40	177.8±12.3	–2.27±0.14	0.216	184.7±9.5	–6.49±0.94	0.419
50	50	185.3±4.2	–6.12±0.15	0.242	192.7±3.7	–9.81±0.78	0.281
40	60	218.2±2.5	–8.51±0.15	0.214	231.5±2.7	–16.36±3.43	0.339
30	70	242.5±2.8	–10.56±0.42	0.167	258.3±4.5	–20.84±4.01	0.557
20	80	274.1±2.1	–11.62±0.63	0.188	241.7±3.6	–14.67±0.8	0.443
10	90	281.6±2.7	–16.72±1.07	0.236	225.3±2.8	–15.32±0.4	0.518
0	100	184.1±29	–4.99±0.61	0.207	261.8±3.9	–6.01±0.43	0.197

Table S6 MSLNP particle size, zeta potential, and DPI data associated with different $HGL_{(DEEN\text{-}\beta CDs)}$/EYPC ratios

HGL (DEEN-βCDs)	EYPC (mol%)	Undiluted			Tenfold diluted		
		Particle size (nm)	Zeta potential	Maximum PDI	Particle size (nm)	Zeta potential	Maximum PDI

(mol%)			(mV)			(mV)	
90	10	268.7±18.2	+8.12±4.15	0.712	1924.3±96.7	–3.45±1.28	1.00
80	20	195.4±3.2	+3.87±3.25	0.521	1134.8±59.3	–2.81±1.95	0.881
70	30	176.3±6.4	–1.24±0.12	0.327	498.7±104.7	–4.17±1.58	0.713
60	40	169.9±5.9	–3.05±0.21	0.194	214.6±21.2	–7.83±1.12	0.397
50	50	181.2±5.4	–5.91±0.22	0.228	228.3±14.1	–10.45±0.85	0.338
40	60	200.1±3.3	–9.27±0.18	0.251	245.9±9.9	–18.94±3.15	0.375
30	70	240.3±6.1	–12.38±0.51	0.203	280.2±15.2	–22.67±4.35	0.592
20	80	263.4±9.8	–13.95±0.72	0.241	266.8±14.7	–17.84±1.2	0.487
10	90	292.1±3.5	–18.06±1.25	0.275	258.4±19.4	–16.79±0.7	0.554
0	100	184.1±29	–4.99±0.61	0.207	261.8±3.9	–6.01±0.43	0.197

Table S7 MSLNP particle size, zeta potential, and DPI data associated with different $HGL_{(AEP\text{-}\beta CDs)}$/EYPC ratios

HGL $_{(AEP\text{-}\beta CDs)}$ (mol%)	EYPC (mol%)	Undiluted			Tenfold diluted		
		Particle size (nm)	Zeta potential (mV)	Maximum PDI	Particle size (nm)	Zeta potential (mV)	Maximum PDI
90	10	--	--	--	--	--	--
80	20	--	--	--	--	--	--
70	30	--	--	--	--	--	--
60	40	--	--	--	--	--	--
50	50	684.2±103.5	+13.85±1.63	0.741	--	--	--

40	60	455.3±73.1	+12.08±5.42	0.724	--	--	--
30	70	334.6±57.9	+7.83±4.02	0.671	--	--	--
20	80	238.1±37.5	+2.15±1.47	0.503	--	--	--
10	90	182.7±18.4	–1.92±0.15	0.358	384.2±31.5	–5.12±1.63	0.441
0	100	179.4±2.9	–3.93±0.14	0.169	267.8±6.2	–6.13±0.56	0.291

* No valid data marked as "--"

Table S8 MSLNP particle size, zeta potential, and DPI data associated with different $HGL_{(AEMO-\beta CDs)}$/EYPC ratios

HGL $_{(AEMO-\beta CDs)}$ (mol%)	EYPC (mol%)	Undiluted			Tenfold diluted		
		Particle size (nm)	Zeta potential (mV)	Maximum PDI	Particle size (nm)	Zeta potential (mV)	Maximum PDI
90	10	--	--	--	--	--	--
80	20	--	--	--	--	--	--
70	30	--	--	--	--	--	--
60	40	--	--	--	--	--	--
50	50	--	--	--	--	--	--
40	60	293.7±6.1	–22.91±5.12	0.628	--	--	--
30	70	271.5±5.3	–18.65±1.45	0.521	--	--	--
20	80	252.8±3.9	–16.04±0.93	0.587	--	--	--
10	90	231.9±14.5	–9.24±2.97	0.363	--	--	--
0	100	182.7±3.7	–5.04±0.27	0.222	257.8±6.5	–5.35±0.43	0.313

* No valid data marked as "--"

Table S9 MSLNP particle sizes associated with different HGL/EYPC ratios, and zeta potential at different temperatures and time (PDI < 0.20 for all groups): (a) 60 mol% $HGL_{(EN-\beta CDs)}$; (b) 50 mol% $HGL_{(EN-\beta CDs)}$; (c) 40 mol% $HGL_{(EN-\beta CDs)}$; (d) 40 mol% $HGL_{(DETA-\beta CDs)}$; (e) 30 mol% $HGL_{(TETA-\beta CDs)}$.

Experimental Conditions			Time				
			6 h	12 h	24 h	72 h	7 d
0 ℃	**a**	Particle size (nm)	153.13±0.40	154.36±0.46	156.68±0.53	166.64±3.12	241.84±3.14
		Zeta potential (mV)	+18.94±0.15	+18.52±0.26	+18.00±0.16	+15.44±0.13	+11.53±0.09
	**b**	Particle size (nm)	144.17±0.71	144.00±0.87	144.37±3.2	145.16±1.35	146.89±3.07
		Zeta potential (mV)	+14.12±0.21	+13.87±0.14	+13.95±0.18	+13.71±0.26	+13.64±0.29
	**c**	Particle size (nm)	145.23±1.38	145.47±0.81	145.89±1.55	148.11±0.74	152.14±1.64
		Zeta potential (mV)	+10.14±0.10	+10.09±0.36	+9.87±0.25	+9.74±0.11	+9.28±0.21
	**d**	Particle size (nm)	153.84±1.12	154.00±1.45	154.67±1.60	155.11±1.75	155.80±1.90
		Zeta potential (mV)	+14.63±0.37	+14.10±0.35	+13.75±0.33	+13.54±0.30	+13.36±0.32
	**e**	Particle size (nm)	156.17±1.86	157.02±2.10	162.16±2.55	165.80±2.95	168.41±1.04
		Zeta potential (mV)	+15.43±0.31	+14.81±0.28	+14.29±0.34	+13.49±0.37	13.07±0.71
25℃	**a**	Particle size (nm)	150.46±2.13	152.28±1.91	153.13±2.12	160.87±1.56	223.82±4.38
		Zeta potential (mV)	+19.01±0.82	+18.88±0.48	+18.53±0.02	+16.77±0.31	+14.06±0.53
	**b**	Particle size (nm)	143.51±1.12	143.16±1.75	143.79±1.05	144.83±4.41	146.17±1.46
		Zeta potential (mV)	+13.99±0.84	+14.00±0.17	+13.94±0.12	+13.82±0.42	+13.89±0.66
	**c**	Particle size (nm)	145.04±1.33	145.85±1.99	146.27±0.81	149.37±1.39	150.39±1.23
		Zeta potential (mV)	+10.00±0.49	+10.21±0.27	+9.94±0.34	+9.53±0.21	+9.41±0.38

	**d**	Particle size (nm)	153.12±2.46	153.85±1.98	154.30±1.75	155.17±2.12	157.44±2.55
		Zeta potential (mV)	+14.59±0.43	+13.92±0.37	+13.65±0.41	+13.48±0.35	+13.17±0.39
	**e**	Particle size (nm)	157.54±2.79	158.20±2.95	161.05±3.16	163.80±3.25	168.58±4.25
		Zeta potential (mV)	+15.88±0.47	+15.20±0.43	+14.65±0.49	+13.25±0.38	+12.59±0.36
38℃	**a**	Particle size (nm)	149.72±1.65	151.06±1.79	152.64±0.74	158.43±1.18	238.33±1.85
		Zeta potential (mV)	+18.87±0.49	+18.53±0.76	+18.50±0.85	+17.08±0.79	+15.13±0.69
	**b**	Particle size (nm)	143.55±2.81	143.49±1.67	143.74±2.15	143.99±1.11	144.11±0.97
		Zeta potential (mV)	+14.08±0.27	+14.01±0.06	+13.94±0.41	+13.97±0.23	+13.89±0.14
	**c**	Particle size (nm)	146.12±1.73	145.99±0.58	146.48±0.74	148.64±1.24	151.11±1.36
		Zeta potential (mV)	+9.96±0.03	+9.96±0.08	+9.76±0.54	+9.57±0.43	+9.22±0.19
	**d**	Particle size (nm)	153.24±1.43	154.50±1.55	154.80±1.70	155.15±1.85	156.16±2.00
		Zeta potential (mV)	+14.37±0.51	+13.85±0.48	+13.70±0.45	+12.95±0.43	+12.20±0.40
	**e**	Particle size (nm)	160.38±3.47	163.63±3.51	169.24±2.97	178.37±3.41	219.93±3.84
		Zeta potential (mV)	+14.94±1.33	+14.71±1.14	+13.97±0.97	+12.67±0.84	+10.37±0.74

Table S10 Encapsulation efficiency and drug loading capacity of Rh B and CBD in LNPs, MSLNPs$_{(EN\text{-}\beta CDs)}$, and LNPs$_{(SM102)}$ formulations

Experimental Conditions	Abs	Dilution factor	M_1 (mmol)	M_2 (mmol)	Encapsulation Efficiency (%)	Drug Loading Capacity (%)
MSLNPs$_{(EN\text{-}\beta CDs)}$-loaded Rh B	0.148	500	1.10×10^{-3}	1.6665×10^{-3}	33.99%	21.87%
MSLNPs$_{(EN\text{-}\beta CDs)}$-loaded CBD	0.301	100	3.13×10^{-2}	6.666×10^{-2}	53.04%	18.50%

LNPs-loaded Rh B	0.167	500	1.19×10^{-3}	1.6665×10^{-3}	28.41%	10.01%
LNPs-loaded CBD	0.295	100	3.08×10^{-2}	6.666×10^{-2}	53.84%	16.44%
$LNPs_{(SM102)}$-loaded Rh B	0.174	500	1.23×10^{-3}	1.6665×10^{-3}	26.45%	9.53%
$LNPs_{(SM102)}$-loaded CBD	0.300	100	3.12×10^{-2}	6.666×10^{-2}	53.14%	17.37%

Abbreviations: Rh B, rhodamine B; CBD, cannabidiol.

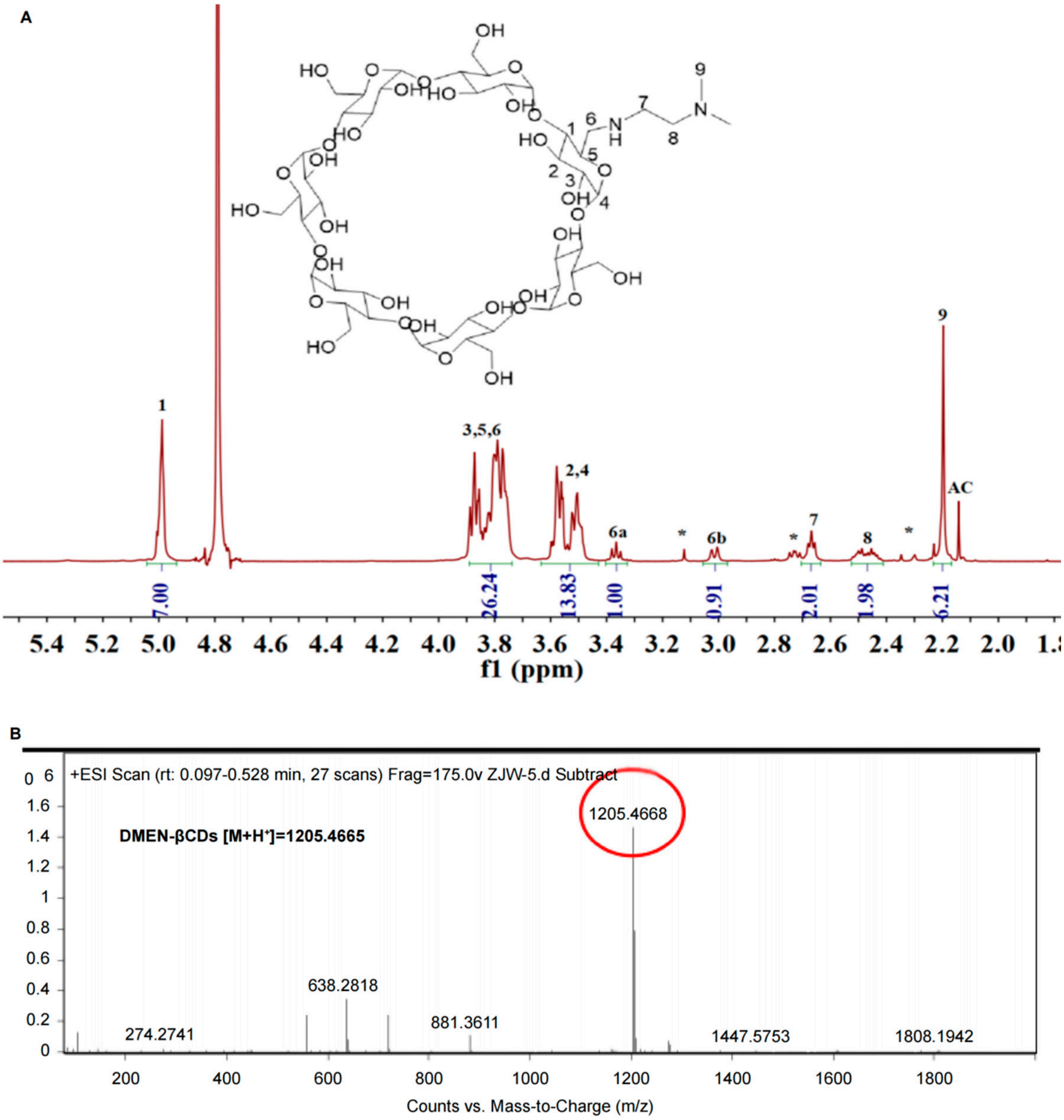

Figure S1 (A) ^{1}H NMR and (B) HR-MS of DMEN-βCDs.

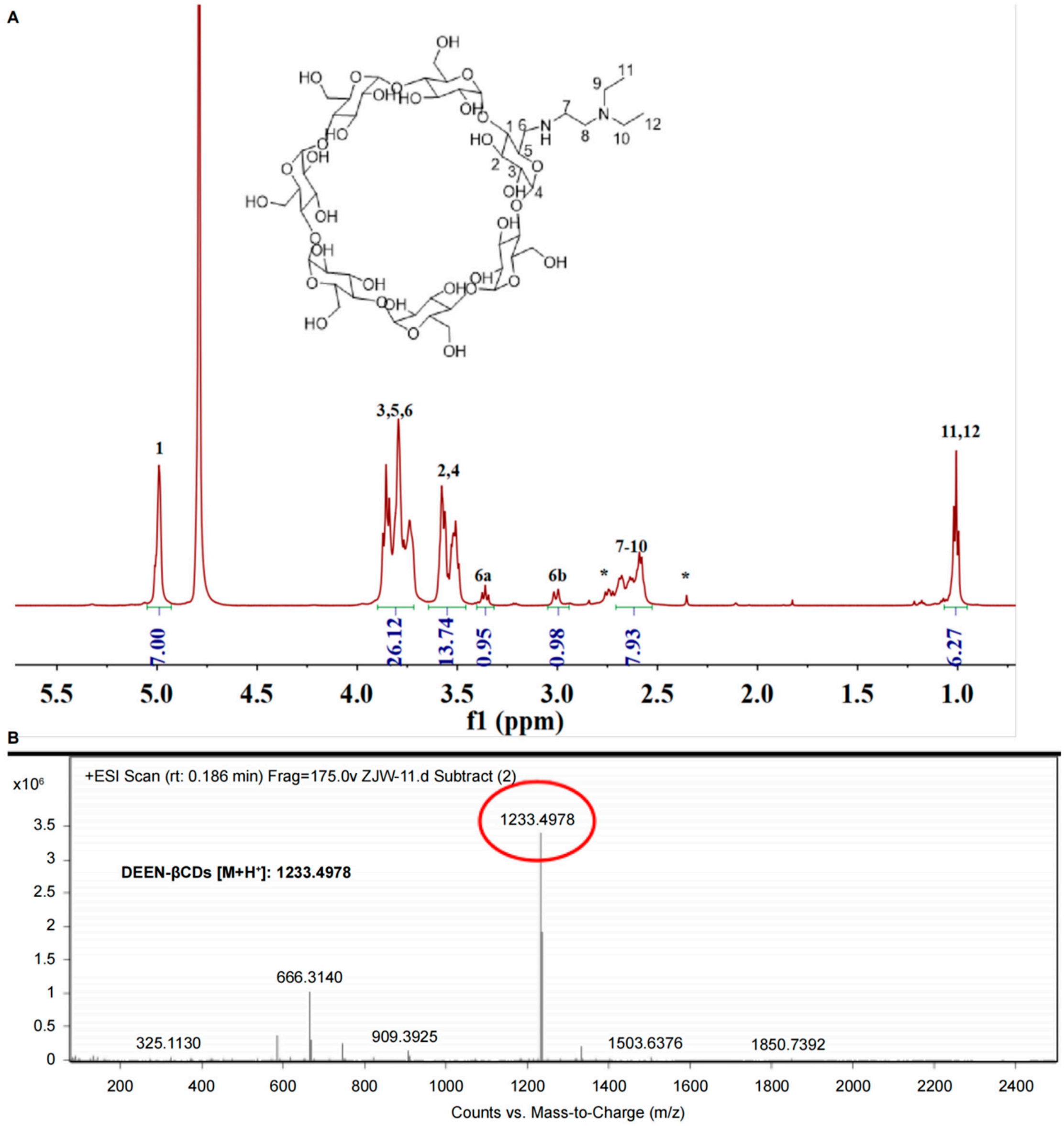

Figure S2 (A) ^{1}H NMR and (B) HR-MS of DEEN-βCDs.

A

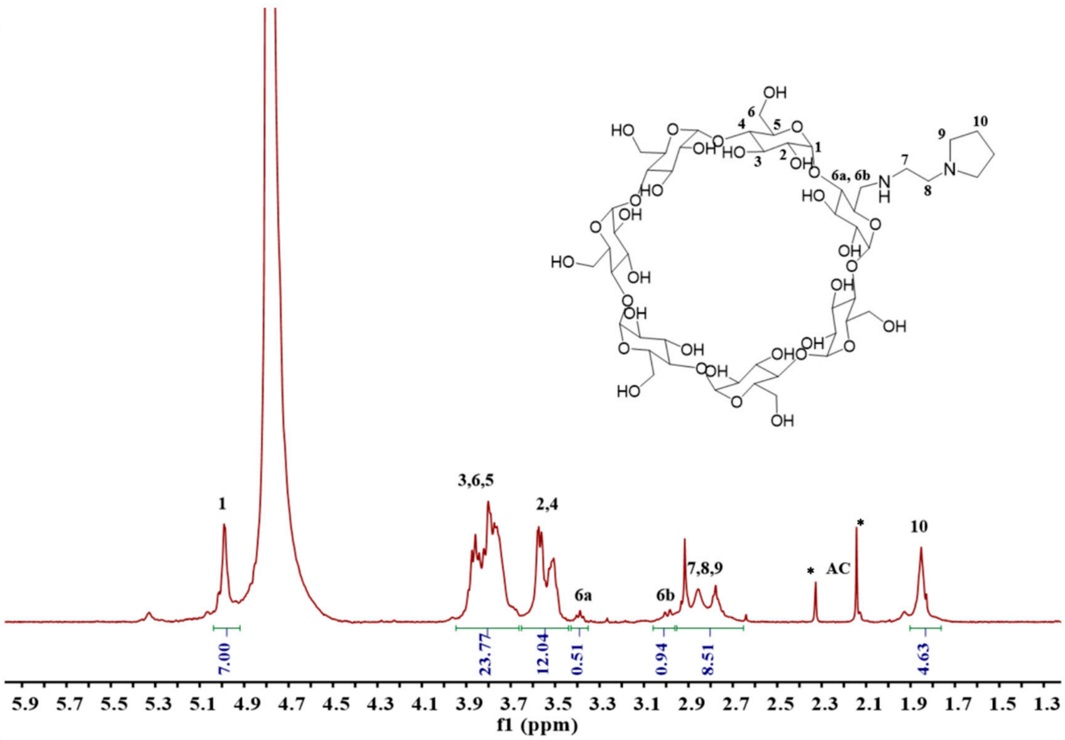

B

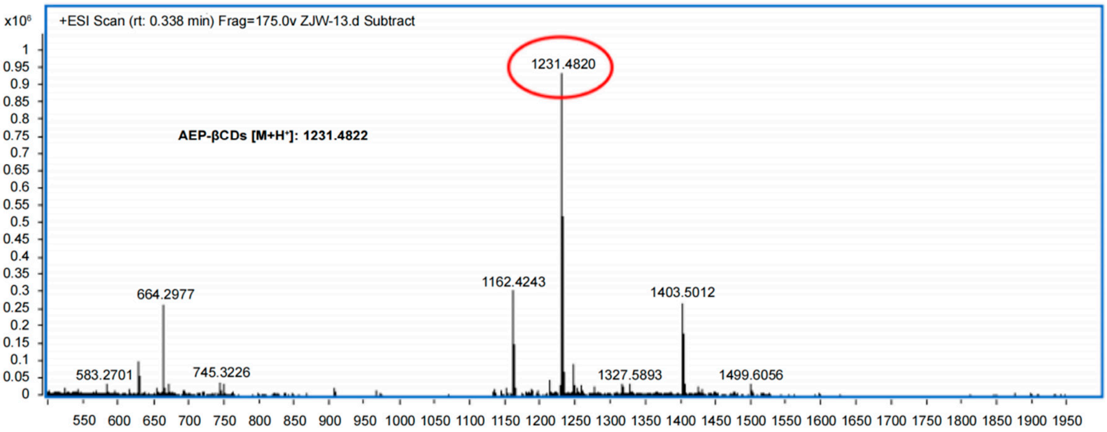

Figure S3 (A) ^{1}H NMR and (B) HR-MS of AEP-βCDs.

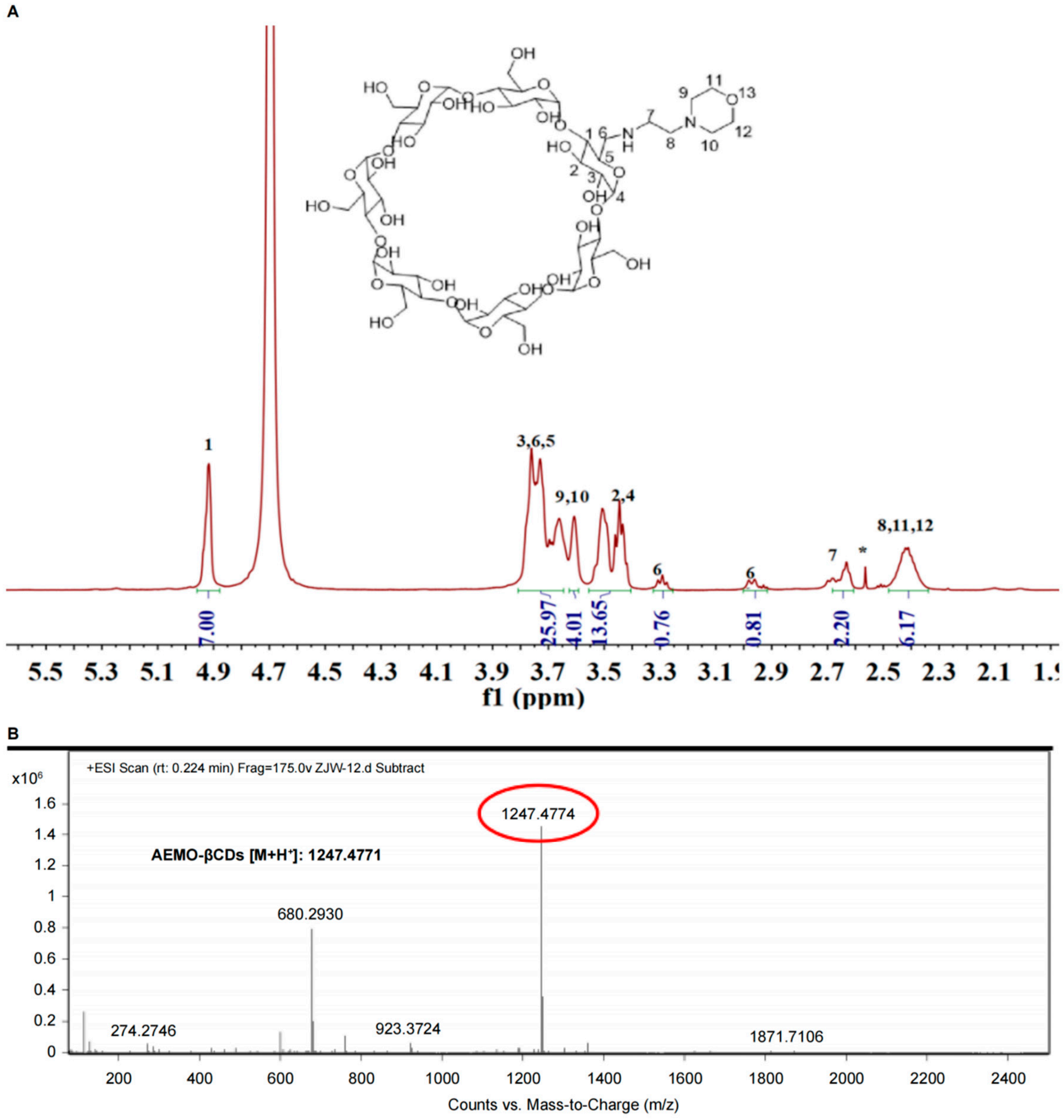

Figure S4 (A) ^{1}H NMR and (B) HR-MS of AEMO-βCDs.

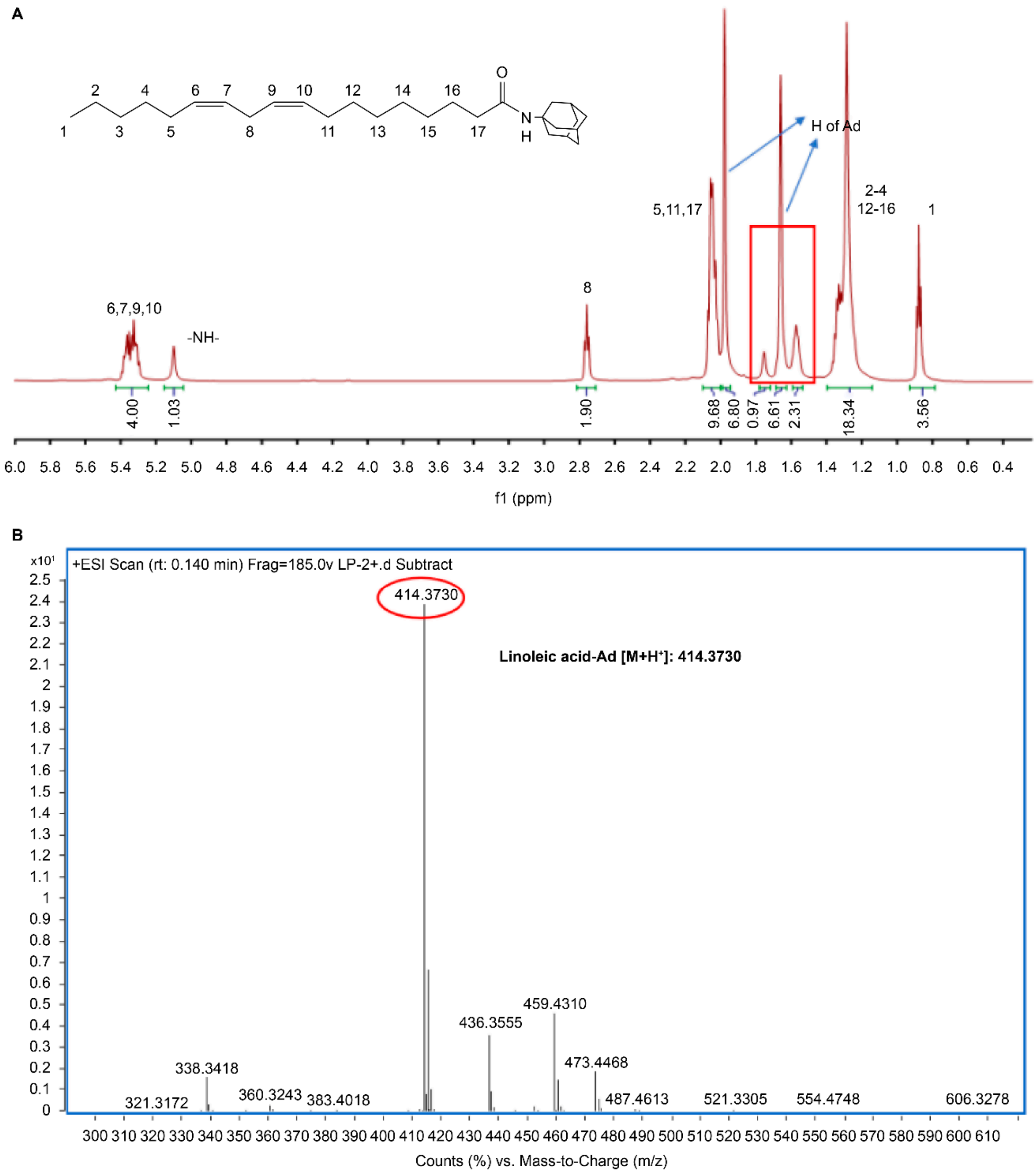

Figure S5 (A) ^{1}H NMR and (B) HR-MS of linoleic acid–Ad.

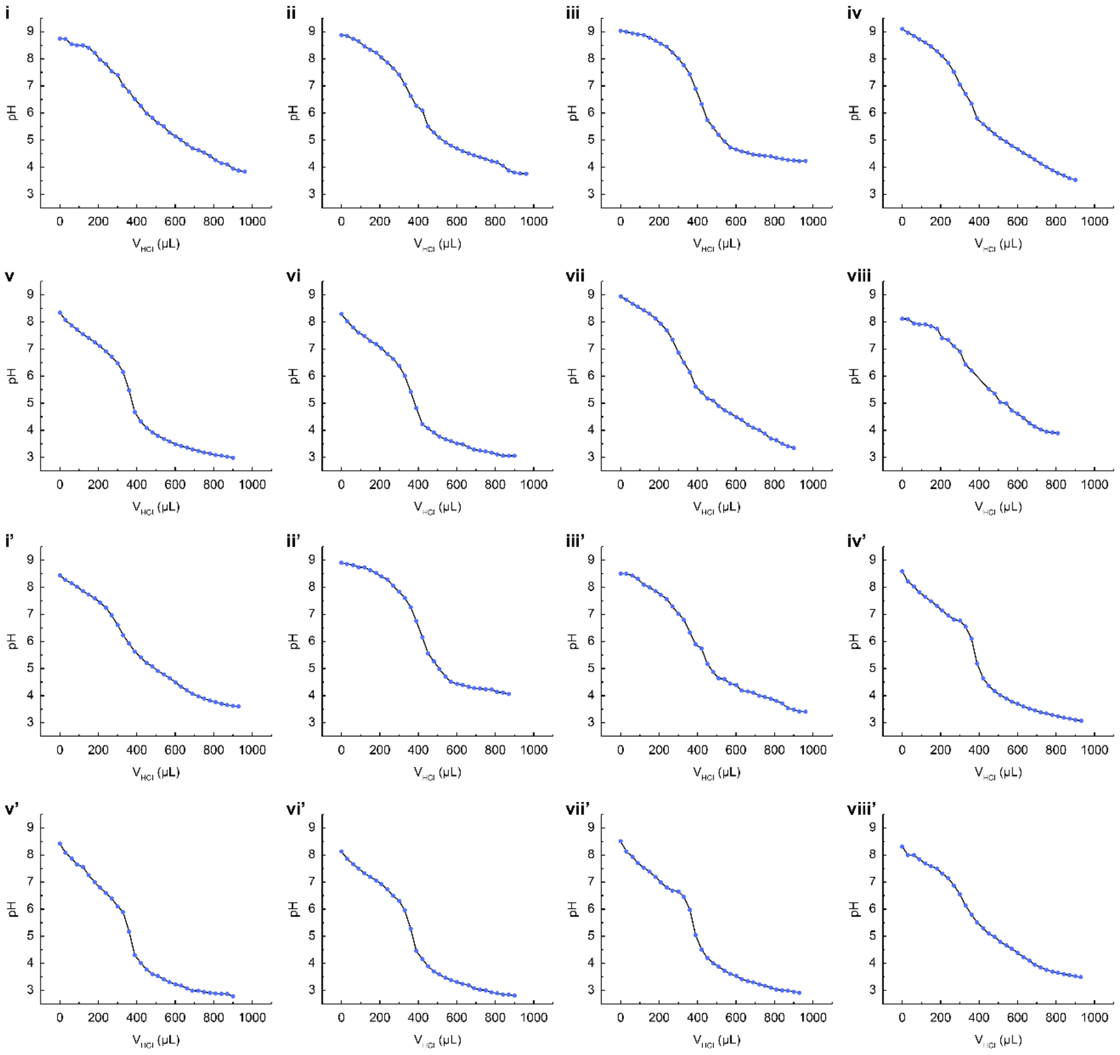

Figure S6 pKa data for eight types of amine β-CDs and their HGLs: i) NH_2-βCDs, pKa 6.549; ii) EN-βCDs, pKa 7.043; iii) DETA-βCDs, pKa 7.369; iv) TETA-βCDs, pKa 7.699; v) DMEN-βCDs, pKa 7.325; vi) DEEN-βCDs, pKa 7.254; vii) AEP-βCDs, pKa 7.247; viii) AEMO-βCDs, pKa 7.675; i') $HGL_{\text{(NH2-βCD)}}$, pKa 5.985; ii') $HGL_{\text{(EN-βCDs)}}$, pKa 6.714; iii') $HGL_{\text{(DETA-βCDs)}}$, pKa 7.213; iv') $HGL_{\text{(TETA-βCDs)}}$, pKa 7.522; v') $HGL_{\text{(DMEN-βCDs)}}$, pKa 7.108; vi') $HGL_{\text{(DEEN-βCDs)}}$, pKa 7.251; vii') $HGL_{\text{(AEP-βCDs)}}$, pKa 7.116; viii') $HGL_{\text{(AEMO-βCDs)}}$, pKa 7.536.

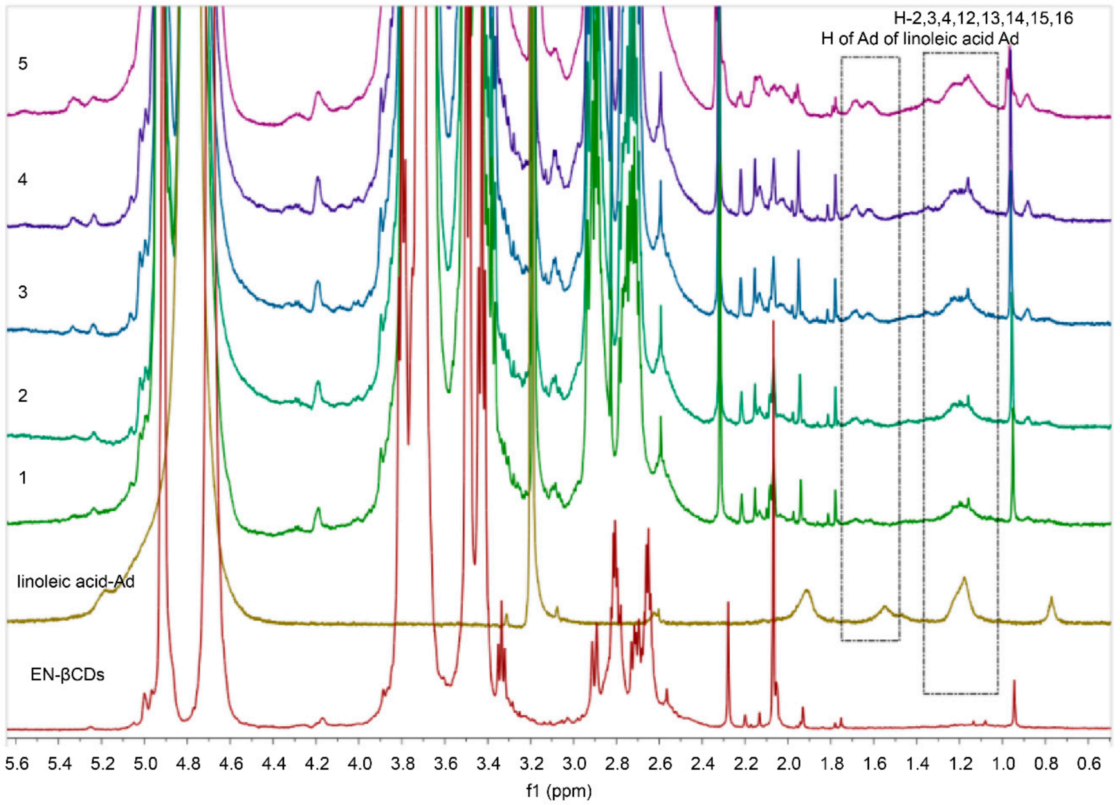

Figure S7 Titration NMR spectra of EN-βCDs and linoleic acid-Ad at different concentrations. From bottom to top, EN-βCDs, linoleic acid–Ad, 10 mg EN-βCDs, 12 mg EN-βCDs, 14 mg EN-βCDs, 16 mg EN-βCDs, and 20 mg EN-βCDs.

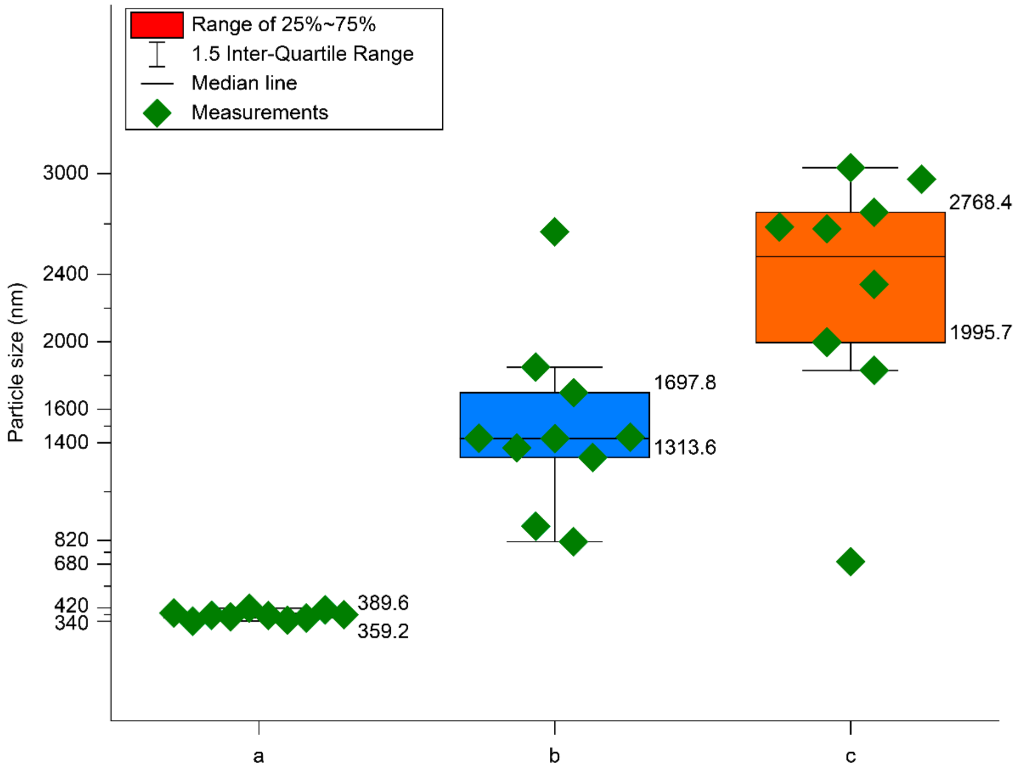

Figure S8 Box plots of particle size measurements for MSLNPs constructed with 100% $HGL_{EN\text{-}\beta CDs}$ under different conditions.

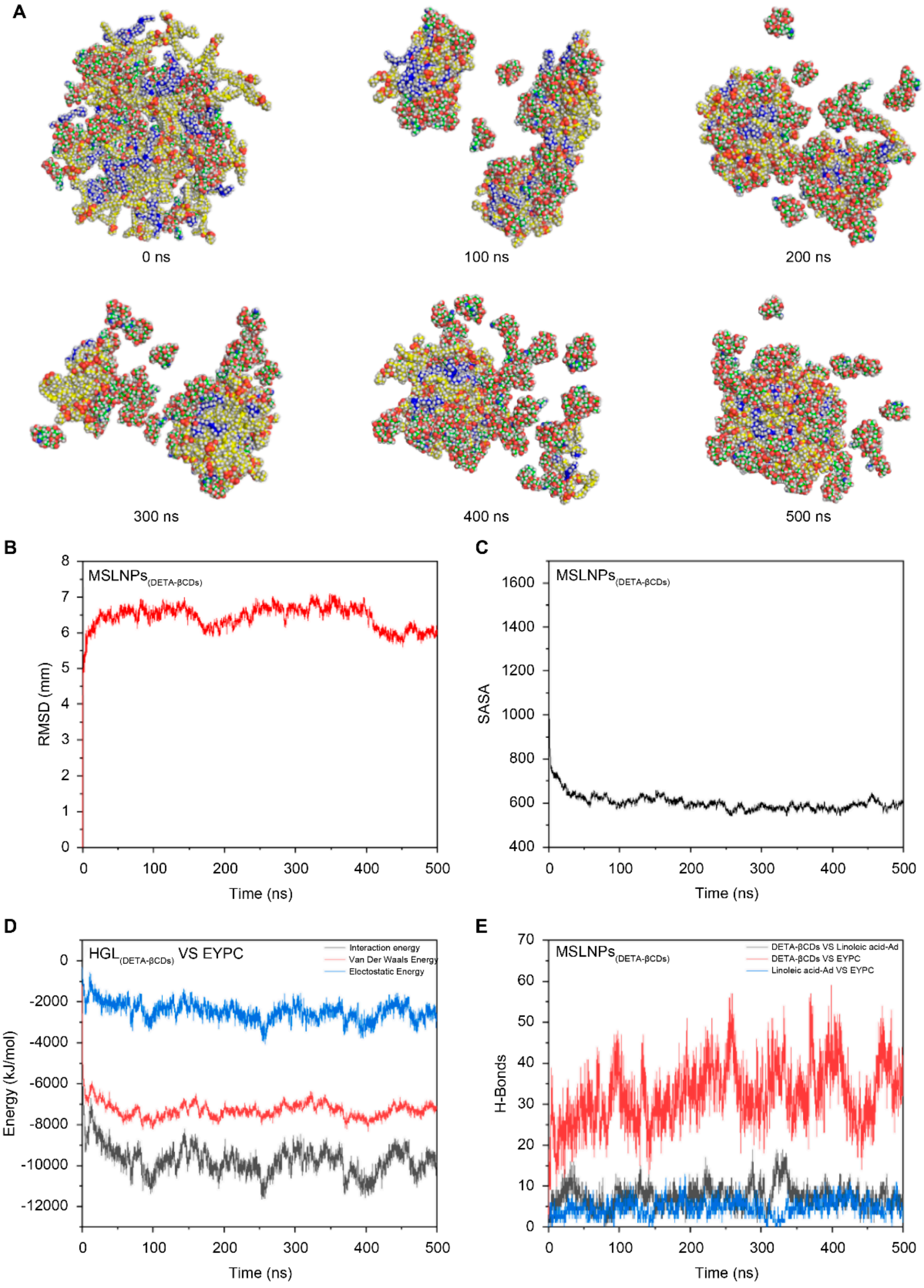

Figure S9 Self-assembly molecular dynamics simulations of MSLNPs$_{(DETA\text{-}\beta CDs)}$. (A) Nanocluster formation of DETA-βCDs, linoleic acid–Ad, and EYPC, with image frames captured at intervals of 100 ns (C of βCDs, C of linoleic acid–Ad, and C of EYPC atoms are marked by green, blue, and yellow spheres, respectively); (B) RMSD of all atoms in MSLNPs$_{(DETA\text{-}\beta CDs}$ system as a function of

simulation time; (C) time-dependent changes in solvent accessible surface area (SASA) in the $MSLNPs_{(DETA\text{-}\beta CDs)}$ systems during simulation; (D) time-dependent changes in electrostatic energy, van der Waals energy, and interaction energy among components in the $MSLNPs_{(DETA\text{-}\beta CDs)\ (HGL \leftrightarrow EYPC)}$. Over 500 ns, the average electrostatic energy was –2457.67±456.33 kJ/mol, average van der Waals energy was –7286.68±410.13 kJ/mol, and average interaction energy was –9744.35±754.00 kJ/mol; (E) time-dependent changes in the number of intermolecular hydrogen bonds in the $MSLNPs_{(DETA\text{-}\beta CDs)}$. DETA-βCDs ↔ linoleic acid–Ad, hydrogen bond counts ranged from 0 to 19, with an average of 7.44±2.78; DETA-βCDs ↔ EYPC, hydrogen bond counts ranged from 0 to 61, with an average of 32.38±7.56; linoleic acid–Ad ↔ EYPC, hydrogen bond counts ranged from 0 to 14, with an average of 4.70±2.03.

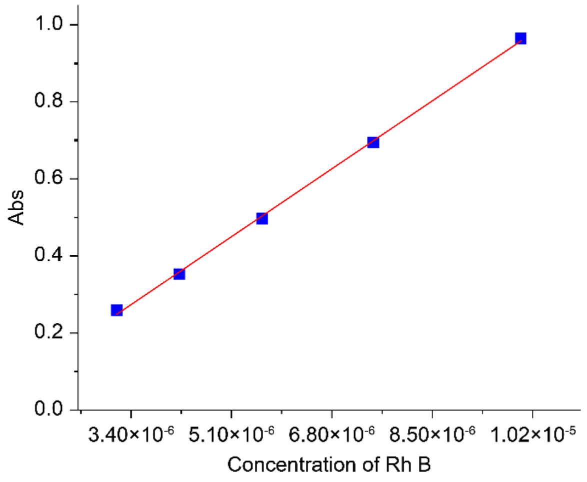

Figure S10 UV standard curve of Rh B, Abs = $103831.15113_{(\text{Concentration of Rh B})}$–0.08053, R^2 = 0.9992.

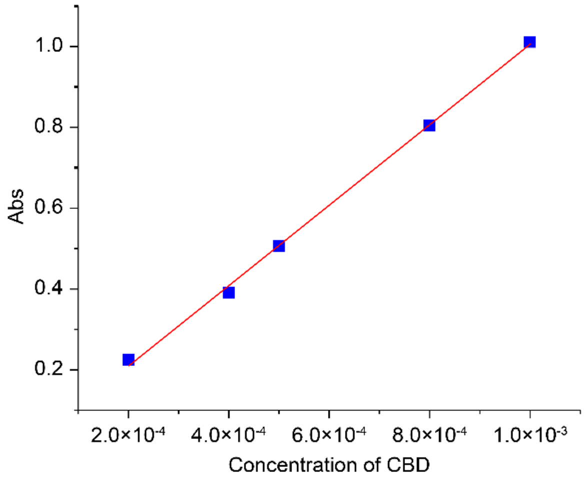

Figure S11 UV standard curve of CBD, Abs = 994.46078$_{\text{(Concentration of CBD)}}$–0.01061, R^2 = 0.9986.

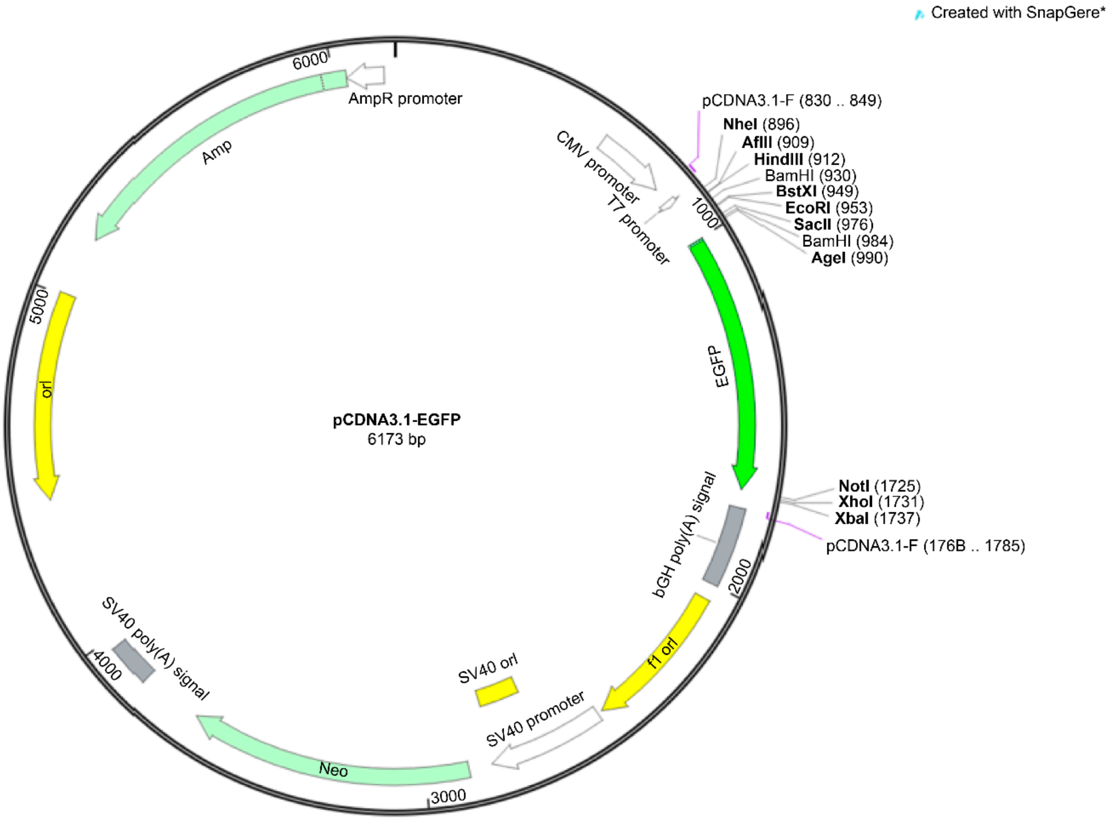

Figure S12 Sequence structure of pcDNA3.1-EGFP.

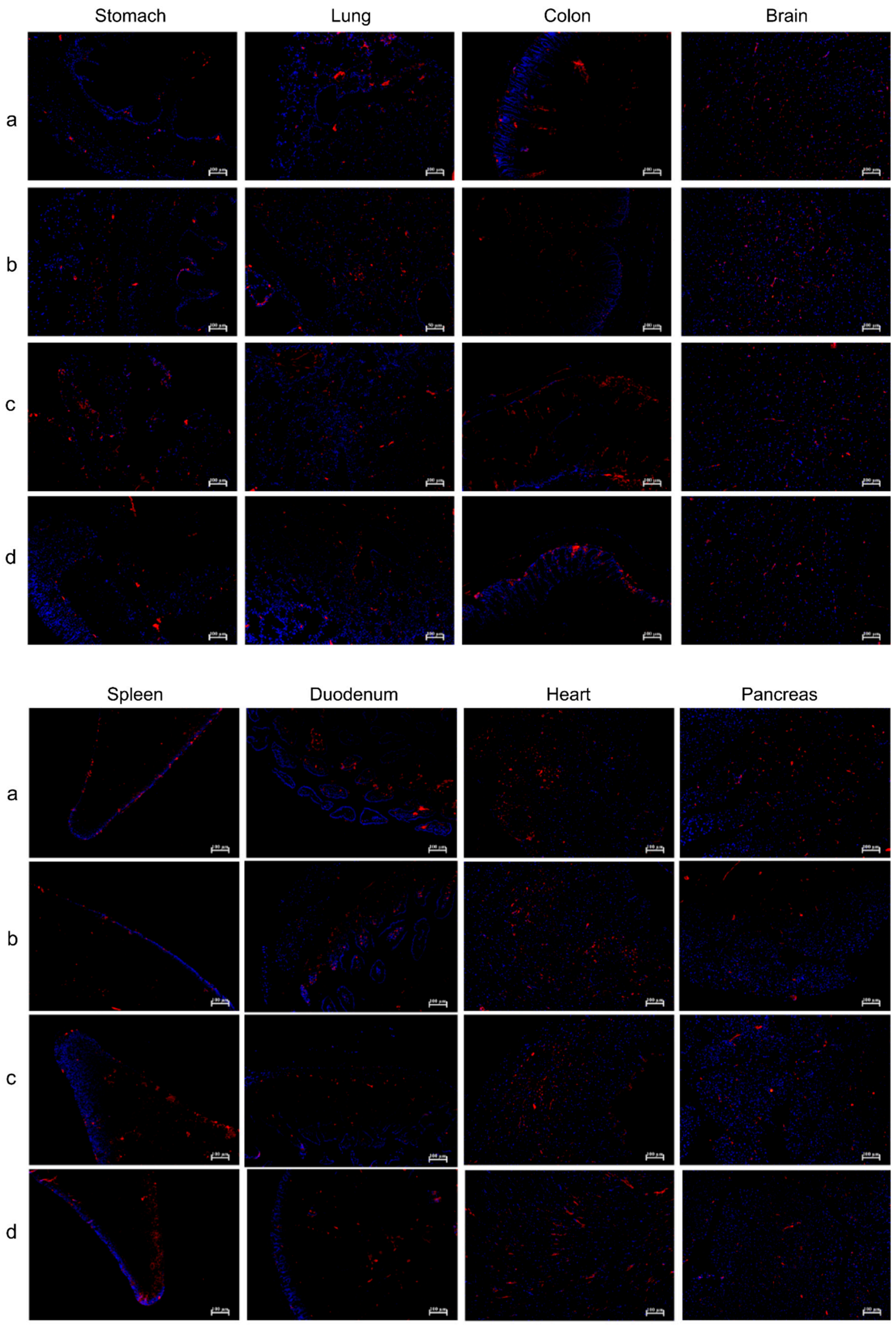
Stomach
Lung
Colon
Brain
a
b
c
d
Spleen
Duodenum
Heart
Pancreas
a
b
c
d

Figure S13 Immunofluorescence results of heart, spleen, lungs, brain, stomach, pancreas, colon, and duodenum in murine. (a: Normal saline group; b: pDNA$_{(EGFP)}$ group; c: LNPs loaded with pDNA$_{(EGFP)}$ group; d: MSLNPs loaded with pDNA$_{(EGFP)}$ group.)

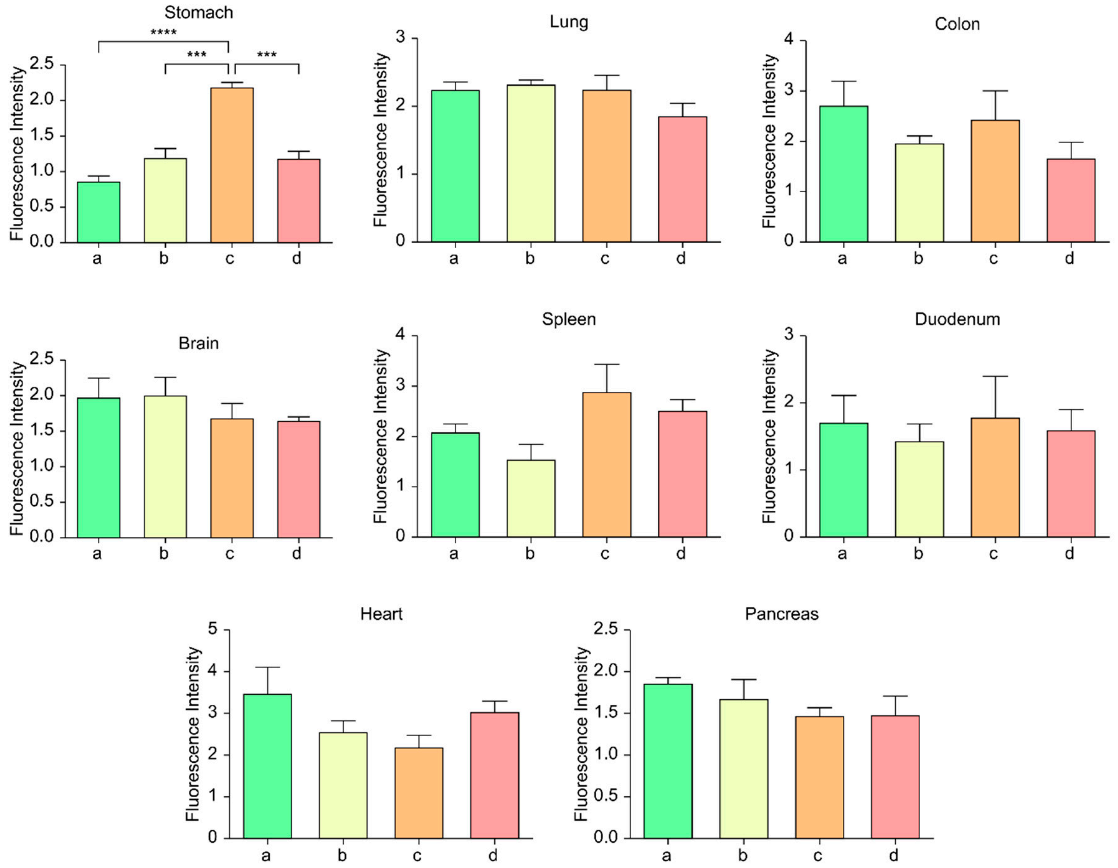

Figure S14 Quantitative analysis of GFP fluorescence intensity across multiple murine organs: (1) stomach; (2) lung; (3) colon; (4) brain; (5) spleen; (6) heart; (7) duodenum; (8) pancreas. (a: Normal saline group; b: pDNA$_{(EGFP)}$ group; c: LNPs loaded with pDNA$_{(EGFP)}$ group; d: MSLNPs loaded with pDNA$_{(EGFP)}$ group (*$P < 0.05$, **$P < 0.01$, ***$P < 0.001$).)
